# Harnessing Amino Acid Modularity for Programmable Function in Covalent Peptide Assemblies

**DOI:** 10.1002/adma.202419941

**Published:** 2025-02-09

**Authors:** Yun‐Mi Hur, Kyoung‐Ik Min

**Affiliations:** ^1^ Department of Biomedical Convergence Science and Technology Kyungpook National University Daegu 41566 Republic of Korea; ^2^ Cell and Matrix Research Institute Kyungpook National University Daegu 41944 Republic of Korea; ^3^ Department of Advanced Bioconvergence Kyungpook National University Daegu 41566 Republic of Korea

**Keywords:** covalent peptide assemblies, multicomponent assembly, sequence–structure–property relationships, single amino acid substitutions, tyrosine

## Abstract

Covalent peptide assembly leverages robust covalent bonds and dynamic non‐covalent interactions to provide enhanced stability and introduce diverse functionalities. Nevertheless, it remains significantly challenging to achieve modular control over the structural diversity and functional complexity while elucidating how specific amino acid sequences contribute to these processes. Here, the systematic encoding of peptide derivative characteristics is demonstrated through amino acid modularity to enable precise control over both the structural diversity and functional complexity in covalent peptide assemblies. By systematically screening single amino acid substitutions in pentapeptides using tyrosine crosslinking, a diverse library of peptide constructs is developed. Each construct is tailored to exhibit distinct properties, including charge repulsion, aggregation‐induced quenching, disassembly behavior, and redox responsiveness. The strategic manipulation of sequence composition, both in individual assemblies and combinatorial systems, enables programmable control over the structural diversity and functional complexity. This approach yields various module‐specific functions, including frustrated growth, hierarchical hollow architecture formation, affinity enrichment, stimuli‐responsive behavior, and fluorescence signal amplification. This work establishes a framework for the design of modular peptide materials with programmable functionalities, advancing the development of next‐generation multicomponent peptide assembly technologies characterized by unprecedented complexity and adaptability.

## Graphical abstract

Covalent peptide assembly integrates the robustness of covalent bonds with the adaptability of dynamic non‐covalent interactions. Systematic amino acid substitution screening yields programmable peptide frameworks, facilitating the advanced engineering of features such as frustrated growth, hierarchical hollow architectures, affinity enrichment, stimuli‐responsive behavior, and signal amplification. This approach revolutionizes multicomponent peptide assembly, offering enhanced complexity, versatility, and multifunctional potential.

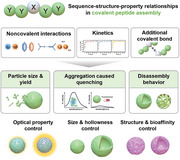

## 1 Introduction

Self‐assembly in biological systems employs various building blocks to perform intricate functions.^[^ ^1^ ^]^ Inspired by these natural processes, chemists have developed peptide self‐assembly systems whereby rationally‐designed peptide building blocks spontaneously form nanostructures via noncovalent interactions such as hydrogen bonding, electrostatic forces, aromatic *π*–*π* stacking, and van der Waals interactions.^[^ ^2^, ^3^ ^]^ Peptides are particularly attractive as building blocks due to their inherent biocompatibility, chemical and structural diversity, robustness, and scalability. Additionally, the direct correlations between peptide sequences and their specific properties and functionalities make the peptides promising candidates for the design of functional nanomaterials in fields such as materials science, engineering, medicine, and biology.^[^ ^2^ ^]^ Understanding the relationship between amino acid composition and the resulting structural and functional characteristics of the peptide assemblies is crucial for tailoring materials for specific applications. However, traditional approaches to peptide design often rely on generating minimalistic peptide sequences derived from protein fragments^[^ ^4^ ^]^ or on inefficient and costly trial‐and‐error methods, both of which lack systematic control.^[^ ^5^ ^]^ Designing peptides with complex functions by screening all possible amino acids leads to a vast design space (20^N^) that is challenging to fully investigate experimentally, even with short sequences. Therefore, there is a pressing need for a more strategic approach that allows for the precise encoding of functionality through amino acid modularity. Recent advancements in peptide co‐assembly have demonstrated the potential to significantly enhance the functional utility and complexity of peptide‐based materials compared to the single‐component self‐assembly approach.^[^ ^6^ ^]^ However, the dynamic nature of noncovalent interactions makes peptide assembly processes highly sensitive to the molecular composition of their building blocks. Even small changes in a single amino acid significantly alter the noncovalent interactions and overall conformation of the assembled structure.^[^ ^5^, ^7^ ^]^ This variability complicates the prediction and understanding of the structural and physicochemical principles governing multicomponent peptide assemblies, which presents challenges in optimizing the sequence–structure–property relationships, particularly as the functional complexity of supramolecular architectures scales with the number of components.^[^ ^5a,b^ ^]^ Additionally, the inherently fragile and reversible nature of noncovalent interactions often makes them vulnerable to disruption by environmental factors such as pH, temperature, ionic strength, and solvents, which can limit their practical applications.^[^ ^8^, ^9^ ^]^

Covalent peptide assembly leverages robust covalent bonds alongside dynamic noncovalent interactions to enhance both physiological and mechanical stability, while also enabling the integration of functionalities that exceed those achievable through conventional supramolecular approaches.^[^ ^9^, ^10^ ^]^ Typical crosslinking strategies employ reactive groups, such as aldehyde, amino, carboxyl, azido, or alkynyl moieties.^[^ ^11^ ^]^ For example, Stupp et al. developed a hybrid polymerization system by combining covalent condensation reactions between aromatic dialdehydes and aromatic diamines with supramolecular polymerization.^[^ ^12^ ^]^ This approach led to the formation of cylindrical fibers with distinct compartments that allowed the removal and reformation of the supramolecular component, thus enabling the reconstitution of the hybrid fibers. Meanwhile, Yan et al. have developed a variety of functional nanoparticles with both physiological stability and biodegradability, based on covalent self‐assembly between various peptide derivatives and crosslinkers such as genipin or glutaraldehyde.^[^ ^9^, ^13^ ^]^ This straightforward method yielded peptide‐based nanodrugs with enhanced stability, tunable optical properties, and controllable light energy conversion, making them suitable for optical imaging and phototherapy applications. Nevertheless, despite the progress in covalent peptide assembly, modular control over the structural diversity and functional complexity remains largely underexplored. Most studies have focused on simple binary mixtures, which restrict the opportunities to explore broader combinatorial designs. Achieving precise control over these aspects requires high‐level programmability via sequence encoding along with a thorough understanding of the roles of individual components within a minimalistic peptide framework.^[^ ^5^, ^14^ ^]^ By characterizing the distinct roles of individual components and implementing a combinatorial design approach, it is possible to systematically encode a diverse array of functional complexities within peptide assemblies.^[^ ^15^ ^]^

**Figure 1:**
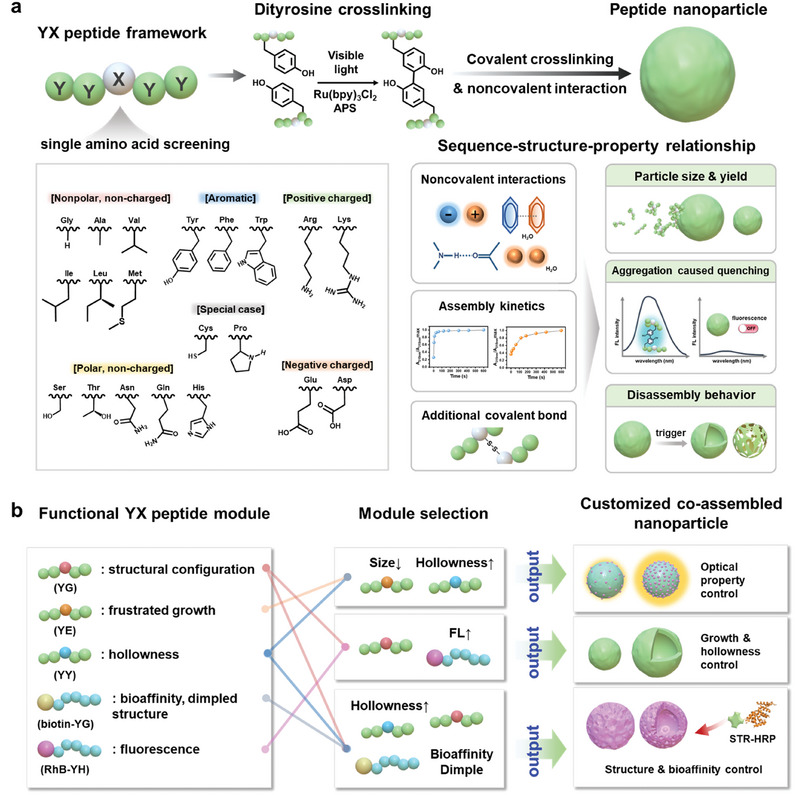
Schematic diagrams illustrating the systematic exploration of covalent *YX* peptide assembly. a) The use of single amino‐acid (*X*) substitution to unveil the sequence–structure–property relationships, including the non‐covalent interactions, assembly kinetics, covalent bonding, particle size and yield, aggregation, and disassembly behavior. b) Modular multicomponent peptide co‐assembly, including the design and selection of functional modules for peptide co‐assembly, thereby facilitating the creation of nanoarchitectures with customizable properties and programmable functions.

Hence, the present study employs the tyrosine‐containing minimalistic peptide framework H_2_N‐Tyr‐Tyr‐X‐Tyr‐Tyr‐COOH (abbreviated as *YX*, where *X* is a canonical amino acid), which promotes the formation of high‐density covalent bonds between adjacent tyrosine residues in proximate peptides, thus enabling a systematic investigation of the sequence–structure–property relationships in covalent peptide assembly (**Figure** **1**a). The assembly process is simplified by strategically harnessing the properties of tyrosine crosslinking while maintaining a streamlined, modification‐free approach, thus enhancing the predictability of the structural and physicochemical properties of the resulting nanoarchitectures. This is particularly beneficial given the inherent variability of noncovalent interactions within multicomponent peptide assemblies. Unlike non‐covalent systems, the present investigation of single amino acid substitutions eliminates the need for complex prediction and control processes, thereby offering a versatile and robust framework for the systematic exploration of sequence–structure–property relationships. This approach allows for the creation of a library of functional peptide constructs and enables the programmable encoding of complex functions by tailoring the composition of multiple components, leading to modular multicomponent peptide assemblies with improved chemical diversity and functional complexity (Figure 1b). In addition, various programmable functions and combinations thereof are demonstrated, including frustrated growth, hierarchical hollow architecture, affinity enrichment, stimuli‐responsive behavior, and fluorescence signal amplification. This work not only enhances understanding of sequence–structure–property relationships in covalent peptide assembly, but also significantly expands the structural and functional possibilities of peptide assemblies.

## 2 Results and Discussion

### 2.1 Covalent Assembly of YX Peptides

**Figure 2:**
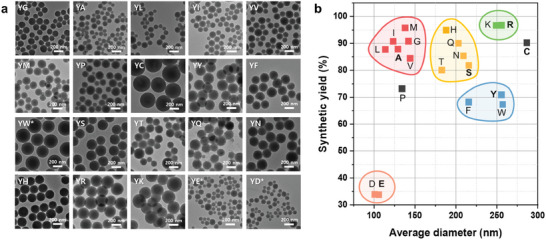
The covalent assembly of 20 different *YX* peptides. a) TEM images of the *YX* nanoparticles. For YW*, a solution of 20:80 (v/v) DMF/pH 10 buffer mixture was used. For YD* and YE*, a final peptide concentration of 2.0 mg mL^−1^ was employed, which is four times higher than the concentration used for the other peptides. b) A plot of the synthetic yield (%) versus average diameter of *YX* nanoparticles, revealing five distinct categories based on the similarities in the central amino acids, excluding *YP* and *YC* (red = nonpolar, green = basic, yellow = polar non‐charged, blue = aromatic, apricot = acidic amino acids).

First, 20 different *YX* peptide nanoparticles were synthesized via one‐step photopolymerization using the well‐known ruthenium (Ru)‐based catalyst (tris(2,2‐bipyridyl)dichlororuthenium(II)hexahydrate, Ru(bpy)_3_Cl_2_).^[^ ^16^ ^]^ Light exposure triggers tyrosine crosslinking in the *YX* peptides, thereby enhancing the non‐covalent interactions that drive in situ self‐assembly. This process increases the hydrophobicity and *π–π* interactions, causing peptide aggregation into globular structures, which eventually form spherical nanoparticles in the aqueous medium.^[^ ^10a,b^ ^]^ Transmission electron microscopy (TEM) images indicate that all of the *YX* peptides are self‐assembled into nanospheres (**Figure** **2**a). Further, the dynamic light scattering (DLS) analysis reveals that all 20 types of *YX* nanoparticles exhibit a monodispersity, with average hydrodynamic diameters depending on the central amino acid, in agreement with the sizes determined from the abovementioned TEM images. (Figure S2, Supporting Information). Meanwhile, the UV–vis analysis reveals a bathochromic shift in the absorbance peaks at ≈285 nm after covalent assembly, which correspond to the π–π* transition in tyrosine residues (Figure S3, Supporting Information). This shift indicates increased electron delocalization across the aromatic rings, which is attributed to the formation of dityrosine bonds during peptide assembly.^[^ ^17^ ^]^ Additionally, all samples exhibit a significant increase in the broad absorbance between 310 and 400 nm after covalent assembly, which is attributed to both the increased opacity due to nanoparticle growth and the formation of dityrosine bonds in the each *YX* nanoparticle.^[^ ^17^ ^]^ Meanwhile, the zeta potential measurements show that all *YX* nanoparticles have negatively charged surfaces, ranging from ≈−20 to −40 mV at neutral pH (Table S1, Supporting Information). Further, the results of a bicinchoninic acid (BCA) assay indicate that the synthetic yields of the *YX* nanoparticles vary depending on the specific central amino acid (Table S1, Supporting Information). Interestingly, the plot of synthetic yield versus average particle diameter for the various *YX* nanoparticles reveals that the peptide sequences fall into five distinct categories based on the similarities of the central amino acids, with the exception of *YP* and *YC* (Figure 2b). Specifically, the peptides with nonpolar amino acids (A, G, I, L, M, V) form small nanoparticles with diameters ranging from 110 to 150 nm and yields of 85–95%. Meanwhile, the peptides with aromatic groups (F, W, Y) produce larger nanoparticles (210–260 nm) but with lower average synthetic yields (65–70%). Peptides with polar amino acids (N, Q, S, T) and deprotonated histidine (H) also result in large particles, with average sizes of 180–220 nm and yields of 80–95%. Peptides with positively charged amino acids (K, R) form large nanoparticles (240–260 nm) with high synthetic yields (>95%). In contrast, peptides containing acidic amino acids (D, E) are undetectable under the same synthetic conditions. However, when the synthetic concentration of *YD* and *YE* peptides is increased fourfold (to 2.0 mg mL^−1^), assembled nanoparticles are observed. Despite the increased concentration, they are the smallest (100–110 nm) and exhibit the lowest yields (≈33%). Consequently, the systematic screening of all 20 amino acids in the peptide derivates has created an extensive design space for tyrosine‐crosslinking‐induced peptide assembly, even with short peptides, thereby facilitating the construction of a library pool for modular covalent assembly.

### 2.2 Characterization and Sequence–Structure–Property Relationships

According to previous works,^[^ ^10a,b^ ^]^ tyrosine crosslinking triggers covalent peptide assembly, thereby promoting the formation of oligomers that aggregate into globular structures in aqueous media, driven by enhanced hydrophobic and *π–π* interactions. The observed variations in synthetic yields and particle sizes arise from the distinct properties of the amino acid substitutions in the peptide framework and differences in molecular weight (fixed at 0.5 mg mL^−1^ rather than mM units). Factors such as amino acid size, overall peptide charge (e.g., *YE* and *YR*), solution pH, and polarity differences (e.g., *YA* and *YS*) significantly influence the assembly process. Peptides with similar physicochemical characteristics tend to exhibit comparable sizes and yields, while peptides with greater differences in polarity or charge result in more varied assembly outcomes. To gain detailed insights into the covalent assembly mechanism and the correlation between sequence, structure formation, and properties of the peptide assemblies, the representative sequences *YA*, *YE*, *YR*, *YS*, and *YY* from each categorized *YX* group were selected for further experiments. Thioflavin T (ThT) staining of five *YX* nanoparticles gives no significant increase in fluorescence signal, thus indicating the absence of β‐sheet secondary structures in these systems (Figure S4, Supporting Information). Meanwhile, the Fourier transform infrared (FT‐IR) spectrum of *YA* reveals a diminished intensity and shift in the characteristic peaks at 1614 cm^−1^ (aromatic ring C─C stretching vibration), 1513 cm^−1^ (aromatic ring C═C stretching vibration), 1230 cm^−1^ (phenolic C─OH bending vibration), 1195 cm^−1^ (phenolic C─OH stretching vibration), and 1139 cm^−1^ (aromatic ring C─H bending vibration) after nanoparticle formation (Figure S5, Table S2, Supporting Information). Notably, the peaks at 838 and 800 cm^−1^ corresponding to the C─H bending vibration and disubstituted benzene in the monomer are seen to have merged to 828 cm^−1^ after covalent peptide assembly. These phenomena indicate electron delocalization in the phenyl groups, which is likely due to either a reduction in the number of aromatic C─H bonds after dityrosine formation or *π–π* interactions of the tyrosine residues, in agreement with the UV–vis results (Figure S3, Supporting Information).^[^ ^18^ ^]^ The FT‐IR spectra of *YY*, *YS*, *YR*, and *YE* exhibit similar trends to those of *YA*, confirming the tyrosine‐crosslinking‐induced peptide assembly across all groups. Meanwhile, the circular dichroism (CD) spectra of five *YX* peptide monomers exhibit two distinct positive peaks at ≈208 and 230 nm, with a weak valley ≈216 nm (Figure S6, Supporting Information).^[^ ^19^ ^]^ After the formation of peptide nanoparticles, the CD signals have almost vanished, which is most likely due to peptide aggregation via tyrosine‐crosslinking, resembling an aggregation‐annihilation CD phenomenon.^[^ ^20^ ^]^

**Figure 3:**
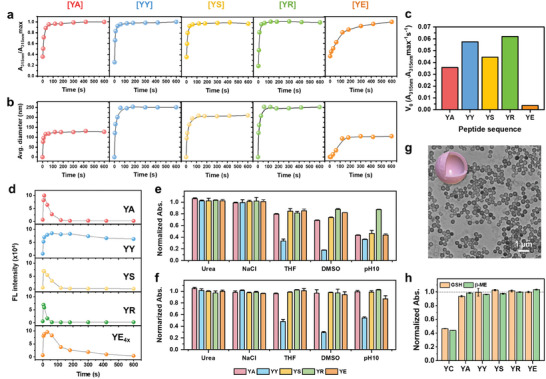
Characterization of representative *YX* peptides. a) The time‐dependent normalized UV–vis absorption profiles at 315 nm, indicating the progress of tyrosine crosslinking and particle formation. b) The change in average diameter over time, as determined by DLS. c) The initial rates of nanoparticle formation, as determined from the initial slope of the normalized UV–vis absorbance at 315 nm over time. d) The variation in fluorescence emission intensity at 400 nm over time, indicating aggregation‐caused quenching. e,f) the normalized absorbance of (e) the incompletely formed *YX* nanoparticles after 2 min of reaction and (f) the fully‐formed *YX* nanoparticles after 10 min of reaction under various conditions for 1 h; 100 mm urea (for hydrogen bonds), 100 mm NaCl (for ionic bonds), 20:80 (v/v) THF/water (for *π–π* interactions), 20:80 (v/v) DMSO/water (for hydrophobic interactions), and pH 10 (for pH effects). g) TEM image of the *YY* hollow nanoparticles, as transformed by the pH 10 buffer. h) The stimuli‐responsive disassembly test of the *YC* nanoparticles under conditions of 1% β‐mercaptoethanol (β‐ME) and 0.5 mm glutathione (GSH) for 1 h, with the Y*A*, *YY*, *YS*, *YR*, and *YE* nanoparticles as negative controls.

The time‐dependent UV–vis absorbance profiles of the *YA*, *YY*, *YS*, and *YR* exhibit an initial logarithmic increase, followed by a plateau within ≈60 s (**Figure** **3**a). These trends align with the DLS results (Figure 3b), indicating that the formation of peptide nanoparticles due to tyrosine crosslinking occurs rapidly in the early stages. Further, the initial rate of nanoparticle growth from the UV–vis kinetics curves followed the order *YR* ≈ *YY* >* YS* > *YA* >>* YE* (Figure 3c). The *YR* peptide exhibits a linear concentration‐absorbance relationship, with a significant change in absorbance even in the lower concentration range of 0–0.4 mg mL^−1^ (Figure S7a, Supporting Information). The presence of the guanidino group gives the *YR* peptide a higher isoelectric point, which creates a near neutral net charge under self‐assembly conditions, enhancing the noncovalent aggregation of oligomers and resulting in larger nanoparticle sizes and higher yields. By contrast, the negatively charged *YE* peptide displays slower assembly kinetics, even when used at four times the concentration of the other peptides. To characterize this slow assembly behavior, the critical aggregation concentration (CAC) was determined from the plots of absorbance versus concentration across different pH conditions (Figure S7b–d, Supporting Information). Under synthetic conditions (pH ≈ 8), the CAC of the *YE* peptide is ≈1.99 mg mL^−1^. At pH 9, however, the increased negative potential of the *YE* peptide hinders self‐assembly, such that no significant increase in absorbance is observed even at a concentration of 3 mg mL^−1^. Conversely, at pH 6, the reduced net surface potential and repulsive forces facilitate assembly, thereby lowering the CAC of the *YE* peptide to 0.62 mg mL^−1^. These results indicate that the slow assembly kinetics of the *YE* stem from an energy penalty due to strong electrostatic repulsion, which impedes efficient assembly. Consequently, both the growth rate and final nanoparticle size of the *YE* peptide are suppressed, thereby suggesting a potential role in inducing frustrated growth within modular covalent peptide assemblies.^[^ ^21^ ^]^

The time‐resolved fluorescence analyses of the five *YX* peptide solutions at 400 nm revealed two distinct stages: i) a rapid initial increase in fluorescence intensity corresponding to dityrosine formation, and ii) subsequent aggregation‐caused quenching (ACQ) as the assembly process progresses (Figure 3d; Figure S8, Supporting Information).^[^ ^22^ ^]^ The peptides *YA, YR*, and *YS* rapidly reach peak fluorescence within 10 s, followed by florescence quenching due to ACQ within 2 min. Although the *YE* also exhibits ACQ, the quenching occurs at a slower rate, which is attributed to the reduced reaction kinetics associated with its acidic amino acid (E), as observed in the abovementioned UV–vis and DLS results (Figure 3a,b). The presence of the negatively charged glutamic acid residue in the *YE* peptide likely slows down the self‐assembly process, leading to a more gradual buildup of aggregated structures and, hence, slower fluorescence quenching. This effect highlights how specific amino acid residues can influence the assembly dynamics and ACQ rates of peptide nanoparticles. Notably, the *YY* peptide solution reaches its maximum fluorescence intensity within 60 s, with only 25.9% of its peak fluorescence being gradually quenched, despite exhibiting rapid logarithmic assembly kinetics. This behavior contrasts with the other peptide solutions, which experience rapid quenching. The purified *YY* nanoparticles display no fluorescence, and fluorescence is exclusively observed in the supernatant after centrifugation (Figure S9, Supporting Information). This result suggests that the *YY* peptide nanoparticles experience ACQ, while residual oligomers containing dityrosine remain in the solution throughout the assembly process. The *YF* peptide exhibits similar behavior, retaining fluorescence in the residual oligomers (Figure S10, Supporting Information). These observations suggest an unanticipated termination of the assembly process.

To understand these phenomena, the fate of the Ru‐containing catalyst Ru(bpy)_3_Cl_2_ in the peptide nanoparticle assembly procedure is investigated. First, it can be noted that the UV–vis absorbance peak at ≈285 nm due to the phenol ring in the aromatic peptide groups exhibits a more pronounced bathochromic shift compared to other peptides following nanoparticle formation (Figure S3, Table S1, Supporting Information). In addition, the metal‐to‐ligand charge transfer (MLCT) band of Ru(bpy)_3_Cl_2_ shifts significantly from ≈454 nm before covalent peptide assembly to 469, 462, and 461 nm after assembly for the *YY*, *YF*, and *YW*, respectively, while the other peptide groups display only minor shifts (≈3 nm) (Figure S11, Supporting Information). This pronounced peak shift is attributed to the increased electron‐donating effects arising from noncovalent interactions between the bipyridyl ligands and the peptides.^[^ ^23^ ^]^ Moreover, the inductively coupled plasma mass spectrometry (ICP‐MS) analysis confirm high levels of Ru encapsulation in the aromatic peptide groups, with 79%, 68%, and 62% Ru being entrapped within the *YY*, *YF*, and *YW* peptide nanoparticles, respectively, while the other peptide groups contain less than 25% (Figure S12, Supporting Information). Further, when supplementary Ru catalyst is introduced at an intermediate stage of the reaction for the *YY* peptide, the fluorescence intensity plots reveal the immediate occurrence of ACQ, leading to complete loss of the fluorescence signal in the solution (Figure S13, Supporting Information). Considering the rapid growth rate and the presence of oligomeric residuals, it is likely that a substantial amount of Ru is trapped within the aromatic peptide nanoparticles during the early stages of the reaction, thus leading to a rapid depletion of free Ru around the assembled nanoparticles in the solution. This, in turn, results in incomplete covalent assembly over the course of the reaction and hinders effective peptide crosslinking. This observation implies that the structural organization and assembly process of nanoparticles formed by *YY* peptides may differ slightly from those of other peptides. This scenario is further supported by investigating non‐covalent interactions through the treatment of five representative *YX* nanoparticles with various disruption factors were used: 100 mm urea for hydrogen bonds, 100 mm NaCl for ionic interactions, 20:80 THF: water (v/v) for *π–π* interactions, 20:80 DMSO: water (v/v) for hydrophobic interactions, and a pH 10 buffer to assess the pH effects (Figure 3e,f).^[^ ^24^ ^]^ The incompletely formed *YX* nanoparticles after 2 min of reaction exhibit negligible changes in absorbance when treated with NaCl and urea. However, their absorbance is significantly decreased upon exposure to THF, DMSO, and pH 10, indicating that *π–π* interactions and hydrophobic forces play a pivotal role in driving the self‐assembly of nanoparticles alongside tyrosine covalent crosslinking. Notably, the *YY* nanoparticles exhibit the highest level of decomposition, implying a distinct structural organization compared to the other *YX* nanoparticles. Moreover, after complete reaction, the nanoparticles formed using *YA, YE*, *YR*, and *YS* exhibit significantly enhanced resistance to the various disruptive agents, whereas the fully‐formed *YY* nanoparticles, composed solely of tyrosine, continue to exhibit a notable decrease in absorbance when exposed to THF, DMSO, and pH 10. This suggests a reduction in covalent interactions within the *YY* nanoparticles, which supports the hypothesis of lower crosslinking density due to the rapid entrapment and dissipation of the free Ru catalyst during the early stages of the reaction. TEM analysis further confirmed that *YY* nanoparticles underwent significant structural transformations upon exposure to THF, DMSO, and pH 10, where the other *YX* nanoparticles are seen to maintain their original morphologies (Figure S14, Supporting Information). Specifically, DMSO induces a destructive disassembly process in the *YY* nanoparticles, resulting in the formation of very thin, covalently stabilized residual shells. Meanwhile, exposure to THF causes shrinkage of the *YY* nanoparticles to form crumpled particles with concave, multicavity surfaces. Notably, after incubation at pH 10, the interiors of the *YY* nanoparticles are selectively disassembled, leading to the formation of hollow nanostructures. This disassembly facilitates the recovery of the intrinsic blue fluorescence provided by their dityrosine bonds, as aggregated peptide compartments are released from the less crosslinked network within the nanoparticles (Figure S15, Supporting Information). When stored in water at room temperature for 14 days, the hollow nanoparticles exhibit no changes in size, along with stable dispersity and stable structural integrity and morphology, showing no significant degradation or collapse (Figure 3g; Figure S16, Supporting Information). Consequently, the relatively low degree of covalent crosslinking in the *YY* peptide nanoparticles opens up opportunities for intriguing morphological transformations, such as the selective disassembly of supramolecular compartments when exposed to disruption agents targeting noncovalent bonds. The disassembly behavior of the *YY* peptide suggests the potential to serve as hollow modules in modular peptide assembly, enabling the template‐free manipulation of hollow nanoarchitectures. This capability significantly enhances the versatility and functional adaptability of the assembly system.

The unclassified peptide *YC* exhibits distinctive properties attributed to its unique thiol group, and forms nanoparticles with an average diameter of ≈286.5 ± 82.7 nm and a yield of ≈87% (Figure 2b, Table S1, Supporting Information). The thiol group is prone to oxidation, thereby facilitating the simultaneous formation of disulfide bonds alongside tyrosine crosslinking. This incorporation of disulfide bonds accelerates the growth rate of the nanoparticles, which allows them to reach 95.3% of their maximum A_315_ _nm_ within 10 s, ultimately leading to the formation of the largest nanoparticles (Figure S17, Supporting information). In addition, the intrinsic fluorescence of dityrosine in the *YC* nanoparticles initially increases over time, followed by ACQ, similar to behavior observed in other *YX* peptides (*X* = A, R, S, and E) (Figure S18, Supporting information). Importantly, the *YC* nanoparticles exhibit disassembly in the presence of GSH and β‐ME, confirming the presence of additional disulfide bonds and a stimuli‐responsive nature (Figure 3h; Figure S19, Supporting information). This result demonstrates that the disulfide bonds in *YC* nanoparticles can be cleaved in response to specific chemical stimuli, thus making the *YC* nanoparticles suitable for controlled degradation and release. The *YC* peptide imparts stimuli‐responsive properties to the peptide assemblies, making them suitable for applications in modular co‐assembly, where precise control over nanoparticle disassembly and functionality is essential for applications such as drug delivery and responsive controlled systems.^[^ ^25^ ^]^

### 2.3 Expansion of Covalent Peptide Assembly to N‐Terminal Modified Peptides

**Figure 4:**
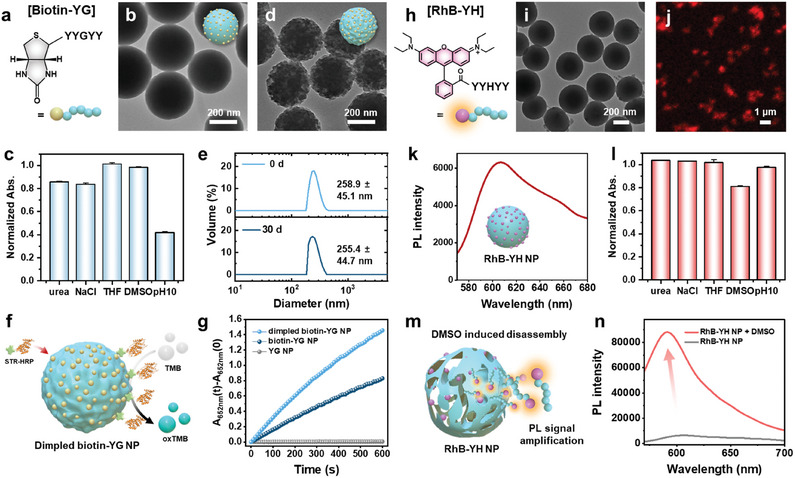
The assembly and characterization of N‐terminal modified peptides. a,b) a schematic representation (a) and TEM image (b) of the biotin‐functionalized *YG* peptide at the N‐terminus. c) The normalized absorbance of the biotin‐*YG* nanoparticles after treatment with various solutions, including 100 mm urea, 100 mm NaCl, 20:80 THF: water (v/v), 20:80 DMSO/water (v/v), and pH 10 buffer. d) TEM image of the biotin‐*YG* nanoparticles after treatment with a pH 10 buffer, indicating that the nanoparticles undergo significant transformation into raspberry‐like morphologies with multiple dimples in response to the pH shift. e) The size distribution of dimpled biotin‐*YG* nanoparticles before (top) and after (bottom) 30 days in aqueous storage, indicating the long‐term storage stability. f) Schematic illustration of the immobilization of HRP on dimpled biotin‐*YG* nanoparticles via biotin‐streptavidin binding, thus enabling the enzymatic oxidation of TMB. g) The time‐dependent variations in absorbance at 652 nm for the spherical biotin‐*YG*, dimpled biotin‐*YG*, and pristine *YG* in the presence of STR‐HRP and TMB. h) Schematic representation of the RhB‐functionalized *YH* peptide at the N‐terminus. i–k) TEM image (i), confocal image (j), and PL emission spectrum under 530 nm excitation (k) of the RhB‐*YH* nanoparticles produced from 1 mg mL^−1^ of peptide monomer. l) The normalized absorbance of the RhB‐*YH* nanoparticles after exposure to various conditions. m) Schematic illustration depicting the fluorescence amplification of the RhB*‐YH* nanoparticles triggered by DMSO‐induced disassembly. n) Comparison of the PL emission of the RhB‐*YH* nanoparticles before (gray) and after (red) the addition of DMSO (50:50 v/v relative to RhB‐*YH* nanoparticle aqueous solution) with a reaction time of 30 s, demonstrating significant PL enhancement as a result of DMSO‐triggered disassembly.

To broaden the functional scope of covalent peptide assembly, we examine the assembly of N‐terminal functionalized peptide derivatives. Specifically, to impart bio‐affinity to the peptide assemblies, the model peptide biotin‐*YG* is selected due to its strong binding affinity and high specificity for the avidin family (**Figure** **4**a).^[^ ^26^ ^]^ The covalent assembly of biotin‐*YG* generates well‐defined nanospheres with an average diameter of 274.6 ± 44.1 nm, which are larger than the pristine *YG* peptide assemblies (Figure 4b; Figure S21a, Supporting Information). Noncovalent interaction experiments reveal a ≈15% decrease in the absorbance of the biotin‐*YG* nanoparticles upon exposure to urea and NaCl, indicating the presence of additional hydrogen bonding and electrostatic interactions that are not detected in the pristine *YG* peptide (Figure 4c; Figures S22, and S23, Supporting Information). Furthermore, after treatment with a pH 10 buffer, the biotin‐*YG* nanoparticles exhibit a ≈60% reduction in absorbance (Figure 4c). This can be attributed to the formation of phenolates, which destabilize noncovalent linkages such as hydrophobic interactions, hydrogen bonding, and electrostatic interactions. Moreover, the TEM image confirms that treatment of the biotin‐*YG* nanoparticles at pH 10 facilitates the breakdown of noncovalent linkages, thus resulting in the formation of dimpled, raspberry‐like nanoarchitectures with multiple bumps (Figure 4d). The selective disassembly process can facilitate the exposure of abundant functional groups, enhancing their potential for affinity‐based functionalization. The dimpled biotin‐*YG* nanoparticles exhibit long‐term storage stability for 30 days, as demonstrated by their consistent hydrodynamic average diameter and preserved morphologies (Figure 4e; Figure S24, Supporting Information).

To further verify the intrinsic binding ability of biotin on the peptide nanoparticles, streptavidin‐conjugated horseradish peroxidase (STR‐HRP) was applied to spherical biotin‐*YG*, dimpled biotin‐*YG*, and pristine *YG* nanoparticles (Figure 4f), and the enzymatic activity was assessed using the chromogenic substrate TMB (3,3′,5,5′‐tetramethylbenzidine). The catalytic conversion was monitored by measuring the absorbance at 652 nm (Figure 4g). Both the spherical and dimpled biotin‐*YG* nanoparticles exhibit time‐dependent increases in absorbance at 652 nm, confirming the successful conjugation of STR‐HRP, while the pristine *YG* nanoparticles show no catalytic activity. Notably, the dimpled, raspberry‐like biotin‐*YG* nanoparticles exhibit enhanced catalytic activity compared to their spherical counterparts. The successful enzyme complexation of dimpled biotin‐*YG* nanoparticles is attributed to the exposure of buried biotin functional groups and the presence of open, accessible compartments that provide abundant binding sites. These features suggest that the biotin‐*YG* peptide has significant potential to impart bio‐affinity and enhance the structural diversity within modular covalent peptide assemblies.

To modulate the optical properties of the *YH* peptide assembly, the corresponding rhodamine B‐functionalized peptide (RhB‐*YH*) was employed (Figure 4h). The covalent assembly of the RhB‐*YH* peptide results in nanoparticles with distinctive photoluminescence (PL) properties and an average diameter of 325.3 ± 105.9 nm (Figure 4i,j; Figure S21b, Supporting Information). A comparison of the PL spectra of RhB‐*YH* and *YY* peptides upon excitation at 450 and 530 nm reveals significantly different optical properties (Figure S25, Supporting Information). Upon excitation at 450 nm (spin‐allowed ^1^MLCT of Ru complexes), the RhB‐*YH* nanoparticles exhibit extremely faint PL emission at 610 nm, whereas the *YY* nanoparticles display significantly stronger emission intensity. These observations align with ICP‐MS results, which indicate Ru entrapped percentages of 0.86% in RhB‐*YH* and 79% in *YY* nanoparticles. However, upon excitation at 530 nm, which corresponds to the promotion of an electron from the ground state of RhB (^1^RhB) to its singlet excited state (^1^RhB*), the RhB‐*YH* nanoparticles do not display the intrinsic RhB emission peak at 590 nm. Instead, they emit the characteristic luminescence of Ru(bpy)_3_Cl_2_ at 610 nm (Figure 4k; Figure S25b, Supporting Information). Notably, the luminescence emitted by RhB‐*YH* nanoparticles at 610 nm is approximately twice as intense as that emitted by *YY* nanoparticles, despite the latter having a higher Ru content. This phenomenon is attributed to an intersystem crossing process, whereby singlet‐to‐triplet energy transfer occurs from the ^1^RhB* excited state at the peptide N‐termini to the lowest ^3^MLCT excited state of the Ru complex.^[^ ^27^ ^]^ These findings confirm the successful incorporation and distribution of RhB within the peptide assemblies.

Noncovalent interaction experiments indicate a decrease in the absorbance of the RhB‐*YH* peptide nanoparticles in the presence of DMSO, indicating the potential for stimuli‐responsive disassembly of hydrophobic interactions in the RhB‐*YH* peptide nanoparticles (Figure 4l). Upon DMSO addition, partial disassembly of the RhB‐*YH* nanoparticles results in significant PL amplification at 590 nm, which is attributed to the radiative relaxation of the ^1^RhB* excited state in the dissolved peptide building blocks under 530 nm excitation (Figure 4m,n; Figure S26, Supporting Information). These energy transfer‐based luminescence behaviors and stimuli‐responsive PL amplification properties significantly enhance the optical complexity of the covalent peptide assemblies, demonstrating their potential for advanced optical and stimuli‐responsive applications.

### 2.4 Modular Multicomponent Assembly of YX Peptides

**Figure 5:**
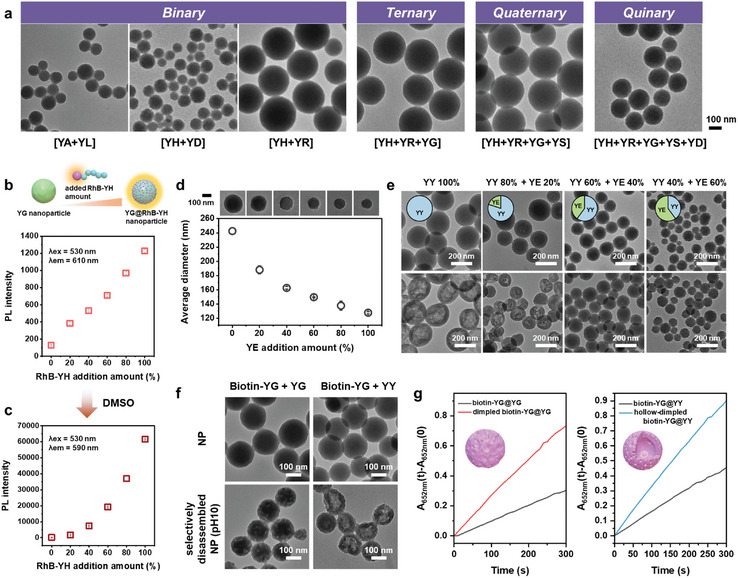
The modular multicomponent assembly of *YX* peptides. a) TEM images of the co‐assembled *YX* nanoparticles incorporating binary to quinary peptide modules. b) PL intensity at 610 nm of RhB‐*YH*@*YG* co‐assemblies, where RhB‐*YH* was incorporated at different concentration ratios (0–100% w/w relative to a fixed concentration of *YG* (0.5 mg mL^−1^)) with excitation at 530 nm. c) The PL amplification of the RhB‐*YH*@*YG* co‐assemblies (excitation at 530 nm, emission at 590 nm) after addition of DMSO (50:50 (v/v) relative to RhB‐*YH*@*YG* aqueous solution), showing signal enhancement upon disassembly triggered by DMSO. d) The TEM images and average diameters of the *YY*@*YE* co‐assemblies formed by adding various *YE* concentrations (0–100% (w/w) relative to *YY*) to a constant concentration of *YY* (0.5 mg mL^−1^), demonstrating a negative size correlation with *YE* concentration. e) TEM images showing the *YY*@*YE* nanoparticles synthesized by varying the compositional ratio of *YY*:*YE* (100:0, 80:20, 60:40, and 40:60 w/w) with a final concentration of 0.5 mg mL^−1^ (top row) and their structural transformations following incubation in pH 10 buffer for 10 min (bottom row). f) TEM images showing biotin‐*YG*@*YG* and biotin‐*YG*@*YY* hybrid nanoparticles before and after selective disassembly via treatment with a pH 10 buffer for 10 min. g) The comparative catalytic activities of structure‐controlled biotin‐*YG*@*YG* and biotin‐*YG*@*YY* nanoparticles following STR‐HRP binding.

Multicomponent peptide co‐assembly offers a transformative approach to enhancing both the structural diversity and chemical complexity of nanoassemblies, effectively addressing the functional constraints of traditional single‐peptide assembly systems. By systematically investigating the sequence–structure–property relationships across diverse peptide derivatives, we developed a comprehensive library of functional peptide modules (Figure 1b). To demonstrate the potential of modular assembly, we explored multicomponent peptide co‐assembly using diverse combinatorial libraries. Combinations of two to five distinct peptide modules within a covalent peptide assembly system yielded multicomponent peptide nanoparticles with narrow, monodisperse size distributions (**Figure** **5**a; Figure S27a, Supporting Information). Further, the amino acid analysis verifies the successful incorporation of all input peptides (*YH*, *YR*, *YG*, *YS* and *YD*) into the quinary‐assembled nanoparticles (Figure S28, Supporting Information). Notably, the combination of *YH* with *YR* results in the formation of significantly larger nanoparticles (244.2 ± 45.1 nm) compared to the unimolecular *YH* assembly (188.2 ± 59.7 nm). In contrast, *YD* exhibits a size‐reduction effect in the binary combination *YH *+ *YD*, producing nanoparticles with a smaller average diameter (140.7 ± 30.8 nm) (Figure S27b, Supporting Information). Furthermore, the addition of *YG* to the *YH *+ *YR* binary system (forming *YH *+ *YR *+ *YG*) results in a similar size (245.2 ± 30.8 nm), as does the quaternary combination with *YS* (*YH *+ *YR *+ *YG *+ *YS*, 222.8 ± 43.2 nm). However, the inclusion of *YD* in the quinary peptide assembly (*YH *+ *YR *+ *YG *+ *YS *+ *YD*) reduces the nanoparticle size to 178.4 ± 37.8 nm, demonstrating the dominant size‐reduction effect of *YD*. These findings reveal the dynamic interplay between the different peptide modules, thus demonstrating their collective role in modulating the co‐assembly system.

To evaluate functional control in binary module co‐assemblies, the concentration of the RhB*‐YH* fluorescence module was systematically varied while maintaining a constant *YG* concentration. This covalent assembly of dyad peptides yielded RhB‐*YH*@*YG* hybrid nanoparticles (Figure S29, Supporting Information). Upon excitation at 530 nm, the hybrid nanoparticles exhibit weak PL emission at 610 nm due to energy transfer from ^1^RhB* to the ^3^MLCT of the Ru complex, and the PL intensity increases in proportion with the concentration of RhB‐*YH* peptides (Figure 5b). Addition of DMSO to the RhB‐*YH*@*YG* nanoparticles results in an amplified PL intensity at 590 nm (excitation at 530 nm) depending on the incorporated amount of RhB‐*YH* (Figure 5c), which is attributed to the partial disassembly, releasing RhB‐*YH* peptides.

The ability to modulate nanoparticle size is a cornerstone for tailoring the physicochemical properties across diverse applications.^[^ ^28^ ^]^ However, precise size control within peptide co‐assembly systems remains challenging due to the intricate interplay of assembly mechanisms and kinetics. In the previous section, it was observed that the negatively‐charged *YE* peptide imposes an energy penalty via strong electrostatic repulsion, thereby restricting the assembly process and limiting nanoparticle growth (Figures 2, 3). This finding suggests that the *YE* peptide can act as a growth frustrating factor, effectively enabling size regulation. Hence, the potential for modularly encoding frustrated growth properties within a co‐assembly system was explored by adding various concentrations of *YE* modules to a fixed concentration of *YY* peptides (Figure 5d; Figure S30, Supporting Information). Notably, an inverse correlation emerges between the *YE* concentration and the sizes of the *YE*@*YY* hybrid nanoparticles, even as the overall concentration of peptide building blocks increased. This relationship, driven by *YE* concentration‐dependent kinetics, suggests that the inherent characteristics of amino acid modularity directly influence the co‐assembled outcomes, enabling precise and on‐demand control over the nanoparticle properties (Figure S31, Supporting Information). In addition, the size limiting effect of the *YE* peptide module is successfully demonstrated in the *YA* peptide assembly system as well (Figure S32, Supporting Information).

To achieve the simultaneous modulation of both size and hollowness, the compositional ratios of the hollowness‐inducing *YY* and the size‐controlling *YE* peptides were systematically adjusted. Increasing the proportion of *YE* results in a decrease in nanoparticle diameter from 246.0 ± 68.4 nm for the unimolecular *YY* nanoparticles to 94.5 ± 30.9 nm for the 40:60 *YY*:*YE* composition (Figure 5e; Figure S33, Supporting Information). Selective disassembly of the *YY*@*YE* hybrid nanoparticles under pH 10 conditions indicates that the hollowness and cavity size increase proportionally with the *YY* composition, while *YE* proportions greater than 40% lead to the formation of solid nanoparticles (bottom row of images, Figure 5e). Collectively, these findings highlight the ability to strategically tailor the structural and functional properties of covalent peptide assemblies by modulating the input composition of peptide modules, advancing the design and adaptability of multifunctional nanomaterials.

To further expand the structural diversity of the covalent peptide assembly system, the biotin‐*YG* module (dimple‐forming and bio‐affinity) with either *YG* or *YY* (hollowness‐inducing) peptide modules. The resulting hybrid nanoparticles exhibited narrow size distributions of 198.6 ± 14.1 nm for biotin‐*YG*@*YG* and 172.7 ± 21.2 nm for biotin‐*YG*@*YY* (Figure 5f; Figure S34, Supporting information). Upon selective disassembly under pH 10 conditions, the biotin‐*YG*@*YG* nanoparticles are reconstituted into dimpled, multicavity‐surfaced architectures (Figure 5f), which is consistent with the earlier observations of biotin‐*YG* nanoparticles (Figure 4c). Meanwhile, the biotin‐*YG*@*YY* hybrid nanoparticles are transformed into hollow/dimpled structures at pH 10, reflecting the synergistic contributions of the hollowness‐ and dimple‐forming capabilities of the input peptide modules. In addition, the structure–activity relationship is revealed by the STR‐HRP bio‐affinity performance evaluation of the hybrid nanoparticles (Figure 5g). Both the solid biotin*‐YG*@*YG* and hollow/dimpled biotin‐*YG*@*YY* display increasing absorbances over time, confirming the retention of intrinsic biotin coupling properties after covalent co‐assembly. Notably, the hollow/dimpled biotin‐*YG*@*YY* particles exhibit a 98% increase in enzymatic activity compared to their solid counterparts. This is attributed to the microenvironments provided by their unique architecture, which improves the binding site accessibility.

Taken together, these findings demonstrate the programmable encoding of functionalities in peptide assemblies via multicomponent modular assembly utilizing covalent networks of functional peptide modules. This strategy significantly expands the toolkit for the on‐demand engineering of peptide architectures with tailored properties. Although this study demonstrates the achievement of diverse functionalities via single amino acid substitutions, its potential extends far beyond. By exploring and modularizing biologically functional peptides, this approach could enable the creation of more sophisticated systems that mimic complex protein functions.

## 3 Conclusion

Herein, the sequence–structure–property relationships in covalent peptide assemblies were systematically explored in order to demonstrate how amino acid composition can be strategically manipulated to control nanostructure properties and functions. First, tyrosine crosslinking‐induced covalent assembly was used to screen all 20 amino acids in peptide derivatives and identify their pivotal roles in modulating the nanoparticle size, assembly kinetics, non‐covalent interactions, aggregation‐induced quenching, and disassembly behavior. Key findings included the size‐limiting effects of *YE*, the hollowness‐inducing properties of *YY*, and the stimuli‐responsiveness of the cysteine‐based *YC*, highlighting the tunability of covalent peptide assemblies. Furthermore, the incorporation of N‐terminal modifications such as biotin and RhB revealed the functional adaptability of the peptide assemblies. RhB*‐YH* functioned as an optical property‐regulating and signal amplification module, while biotin‐*YG* induced dimpled structure and enhanced bio‐affinity. In addition, a strategic multicomponent modular assembly was used to significantly expand the structural and functional diversity of peptide assemblies, achieving precise control over the size, morphology, and multicomponent integration. This work establishes a robust framework for the rational design of programmable and customizable nanomaterials, paving the way for advanced systems with potential applications in controlled drug release, stimuli‐responsive systems, signal amplification in biosensors, enzymatic catalysis, and related fields.

## Conflict of Interest

The authors declare no conflict of interest.

## Supporting information



Supporting Information

## Data Availability

The data that support the findings of this study are available from the corresponding author upon reasonable request.
