# Supplementary material for: Harnessing Amino Acid Modularity for Programmable Function in Covalent Peptide Assemblies
Source: Adv Mater. 2025 Feb 9;37(11):2419941. doi: 10.1002/adma.202419941 (PMC11923520; doi:10.1002/adma.202419941)
Supplement: Supplementary file 1 — Supporting Information [file ADMA-37-2419941-s001.docx]

Supporting Information

Harnessing Amino Acid Modularity for Programmable Function in Covalent Peptide Assemblies

Yun-Mi Hur and Kyoung-Ik Min*

Experimental Section

*Materials*: For this study, all of the pristine peptides H_2_N-Tyr-Tyr-X-Tyr-Tyr-COOH (abbreviated as YX, and X is a canonical amino acid, Table S1) and functionalized peptides, including biotinylated and rhodamine B-labeled peptides (biotin-YG and RhB-YH, respectively) were obtained from GL Biochem, with a purity greater than 98%. Chemical reagents, including tris(2,2'-bipyridyl)dichlororuthenium(II) hexahydrate (Ru(bpy)_3_Cl_2_), ammonium persulfate (APS), phosphate-buffered saline (PBS), dimethylformamide (DMF), dimethyl sulfoxide (DMSO), sodium chloride (NaCl), urea, β-mercaptoethanol (β-ME), glutathione (GSH), and thioflavin T (ThT), were purchased from Sigma-Aldrich. Buffers (pH 6, pH 9, and pH 10) were purchased from Samchun Chemical (South Korea). The ready-to-use substrate reagent 3,3',5,5'-tetramethylbenzidine (TMB) was purchased from BD Bioscience. Streptavidin horseradish peroxidase (STR-HRP) was purchased from Invitrogen. Tetrahydrofuran (THF) was purchased from Daejung Chemical and Metals. The bicinchoninic acid (BCA) protein assay kit and UltraPure™ DNase/RNase-free distilled water were purchased from Thermo Fisher Scientific. All reagents were used without further purification.

*Assembly of the YX peptide nanoparticles:* To eliminate preformed assemblies, peptide solutions were freshly prepared in pH 10 buffer at a concentration of 2.5 mg mL^−1^ and used immediately. For peptides with negatively charged residues (YD and YE), higher-concentration stocks (10 mg mL^−1^) were freshly prepared to overcome the strong electrostatic repulsion between monomers. In the case of YW, the concurrent formation of large spheres was observed due to spontaneous non-covalent interactions in the reaction mixture that was prepared in pH 10 buffer without light irradiation (Figure S1). To prevent the formation of such undesirable large particles prior to covalent assembly, YW was dissolved in a 2:8 (v/v) DMF:pH 10 buffer mixture to enhance its solubility. The biotin-YG was also dissolved in the 2:8 (v/v) DMF:pH 10 buffer mixture at a concentration of 2.5 mg mL^−1^, while the RhB-YH was dissolved in DMSO at 5 mg mL^−1^ for unimolecular assembly and at 2.5 mg mL^−1^ for the co-assembly experiments.

In a typical peptide nanoparticle assembly procedure, the reaction solution was prepared by mixing 0.2 mL of the selected peptide solution with 0.6 mL of aqueous APS (10 mM) and 0.2 mL of aqueous Ru(bpy)_3_Cl_2_ (0.85 mM) in a 1 cm quartz cuvette. The mixture was immediately exposed to a white light lamp system (OSRAM, DULUX L LED, 18 W, 2 ea) with cooling fans at room temperature for 10 min. To ensure dityrosine crosslinking as the dominant pathway in this study, oxygen exposure was minimized during assembly and the reaction was completed within 10 min. After light irradiation, the products were collected by centrifugation (Centrifuge 5425, Eppendorf) at 13,500 rpm for 2 min and washed three times with water. The assembled peptide nanoparticles were dispersed in water at the desired concentration for further use. The yield of YX peptide nanoparticles was determined by quantifying the unreacted peptide monomers in the supernatant after centrifugation, using a BCA protein assay kit. Specifically, 25 μL of the supernatant was combined with 200 μL of the BCA working reagent and incubated for 30 min at 25 °C. The concentration of residual peptide monomers was calculated from the absorbance at 562 nm, referencing a standard curve generated from the YX peptide monomers. For the modular multicomponent assembly of YX peptides, stocks of the peptide components were mixed at varying ratios, and the same assembly process was applied. The specific ratios and components are detailed in the corresponding sections of the manuscript and in Table S3.

*Transmission electron microscopy (TEM) imaging:* For TEM analysis, 10 μL of each sample was deposited onto carbon-coated copper grids (FCF-200 Cu, Electron Microscopy Sciences) and incubated for 5 min, followed by blotting to remove excess material. The grids were subsequently rinsed with water and dried at 60 °C in a drying oven for 12 h. The morphological properties of the YX peptide nanoparticles were examined using Bio-TEM (Hitachi, HT 7700).

*Zeta potential and size analysis:* The average size and zeta potential of each peptide assembly were measured using an ELSZ-2000ZS (Otsuka Electronics). For the dynamic light scattering (DLS) analysis, 0.8 mL of each YX peptide nanoparticle solution at an appropriate concentration was loaded in a micro cuvette. For the zeta-potential analysis, 0.1 mg mL^−1^ of each YX peptide nanoparticle solution in a pH buffer was prepared and stabilized for 30 min. The samples were then loaded into quartz capillary zeta cells. The zeta potentials were measured at room temperature, with at least three measurements performed for each experiment.

*Characterizations:* UV–vis absorption spectra were recorded using a NanoDrop 2000c UV–vis spectrometer (Thermo Fisher Scientific). The fluorescence spectra were measured using a Synergy H1 multi-mode plate reader (Biotek). The Fourier transform infrared (FT-IR) spectra were obtained using a Frontier IR spectrophotometer (PerkinElmer). Circular dichroism (CD) measurements were performed with a JASCO J−1500 CD spectrometer equipped with a Peltier thermostatic cell holder and nitrogen purging. The Ru content encapsulated within the YX peptide nanoparticles was quantified via inductively coupled plasma–mass spectrometry (ICP-MS, Perkin Elmer NexION 300X and NexION 2000).

*ThT staining assay:* The β-sheet conformations of the YX peptide nanoparticles were evaluated using ThT staining. For this procedure, a 40-µL aliquot of YX peptide nanoparticle solution (0.5 mg mL^−1^) was mixed with 20 µL of ThT solution (100 µM) and 160 µL of water. The mixture was incubated at room temperature for 1 h. After incubation, the samples were excited at 445 nm, and the fluorescence emission was measured in the 480–640 nm range (bandwidth = 1 nm) using a multimode microplate reader.

*Investigation of noncovalent interactions:* The noncovalent interactions involved in the assembly of YX peptide nanoparticles (X = A, Y, S, R, E, and G), biotin-YG, and RhB-YH were assessed by dispersing the nanoparticles in various solutions, including 100 mM urea, 100 mM NaCl, 20:80 THF:water (v/v), 20:80 DMSO:water (v/v), and pH 10 buffer. The role of disulfide bonds in YC peptide nanoparticle formation was evaluated using 1% β-ME and 0.5 mM GSH as competitive disulfide bond disruptors. For this experiment, 0.5 mg of YC or YX (X = A, Y, S, R, E) nanoparticles were mixed with 1% β-ME or 0.5 mM GSH aqueous solution. After incubation at room temperature for 1 h, changes in solution turbidity were measured at 380 nm using a UV-vis spectrometer.

*Disassembly of YX peptide nanoparticles:* The disassembly-induced morphological changes in the YX peptide nanoparticles were characterized via TEM imaging after exposure to decomposition conditions. These conditions included noncovalent interaction disruptors (20:80 THF:water (v/v), 20:80 DMSO:water (v/v), and pH 10 buffer) and disulfide bond-breaking agents (1% β-ME and 0.5 mM GSH). The disassembly process was facilitated by 3 min of sonication to accelerate and homogenize the reaction, followed by incubation for 10 min. Without the addition of interaction-disrupting reagents, sonication alone did not affect the disassembly process. The nanoparticles were then washed three times with DI water via centrifugation at 13,500 rpm for 2 min per cycle. The nanoparticles were then redispersed in water for further analysis.

*Photoluminescence amplification:* To investigate the photoluminescence (PL) signal amplification mediated by partial disassembly, DMSO was added to RhB-YH nanoparticle aqueous solution (50:50 v/v) and incubated for 30 s. The PL intensity was then measured using a plate reader with an excitation wavelength of 530 nm.

*Streptavidin-HRP binding assay:* Prior to bioaffinity evaluation, the YX nanoparticles were suspended in a 5% BSA solution for 1 h to block the nonspecific binding sites. The nanoparticles were then thoroughly washed with 0.1X PBS buffer. After that, the YX nanoparticles (0.9 mg mL^−1^) were mixed with STR-HRP (0.01 mg mL^−1^) and continuously stirred at room temperature for 2 h. The resulting STR-HRP-bound YX nanoparticles were collected by centrifugation and washed three times with 0.1X PBS to remove any unbound STR-HRP. For the catalytic activity analysis, 200 μL of TMB substrate solution was added to 10 μL of STR-HRP-bound YX nanoparticles (0.9 mg/mL). The catalytic oxidation of TMB was monitored in real time by measuring the absorbance at 652 nm using the scanning kinetic mode of a UV–vis spectrophotometer.


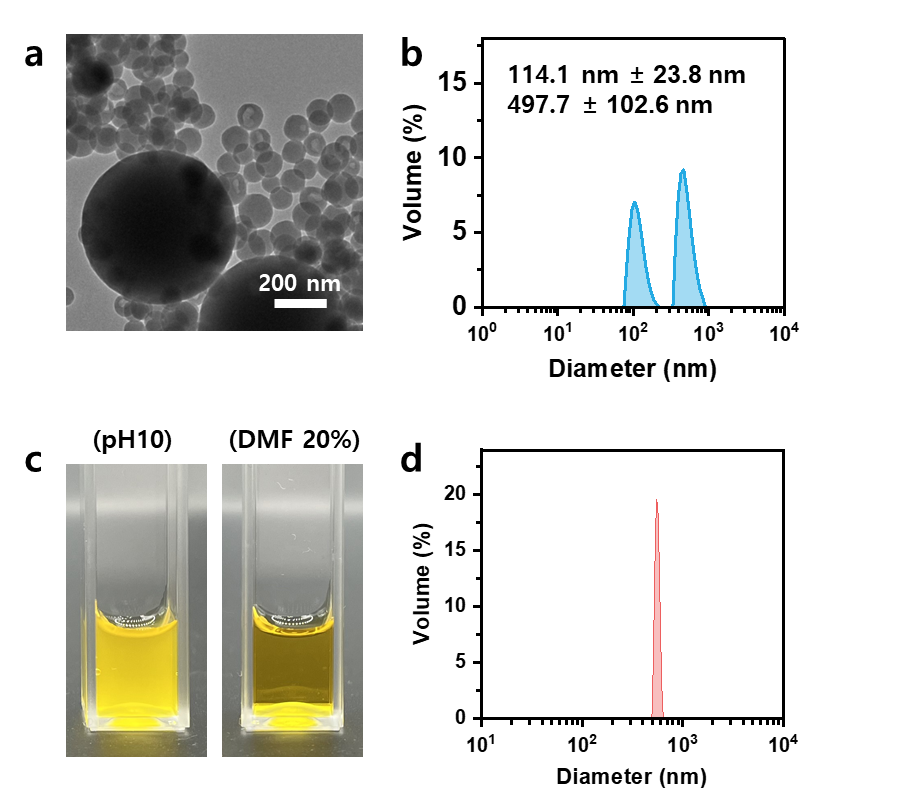


**Figure S1.** (a and b) A TEM image (a) and DLS analysis (b) of the YW peptide nanoparticles prepared in a pH 10 buffer solution, indicating the concurrent formation of large particles. (c) A visual comparison of the reaction mixtures prepared in pH 10 buffer (left) and 2:8 (v/v) DMF:pH 10 buffer mixture (right) before assembly. (d) The DLS analysis of the reaction mixture in pH 10 buffer prior to covalent assembly, showing the presence of non-covalent aggregates.


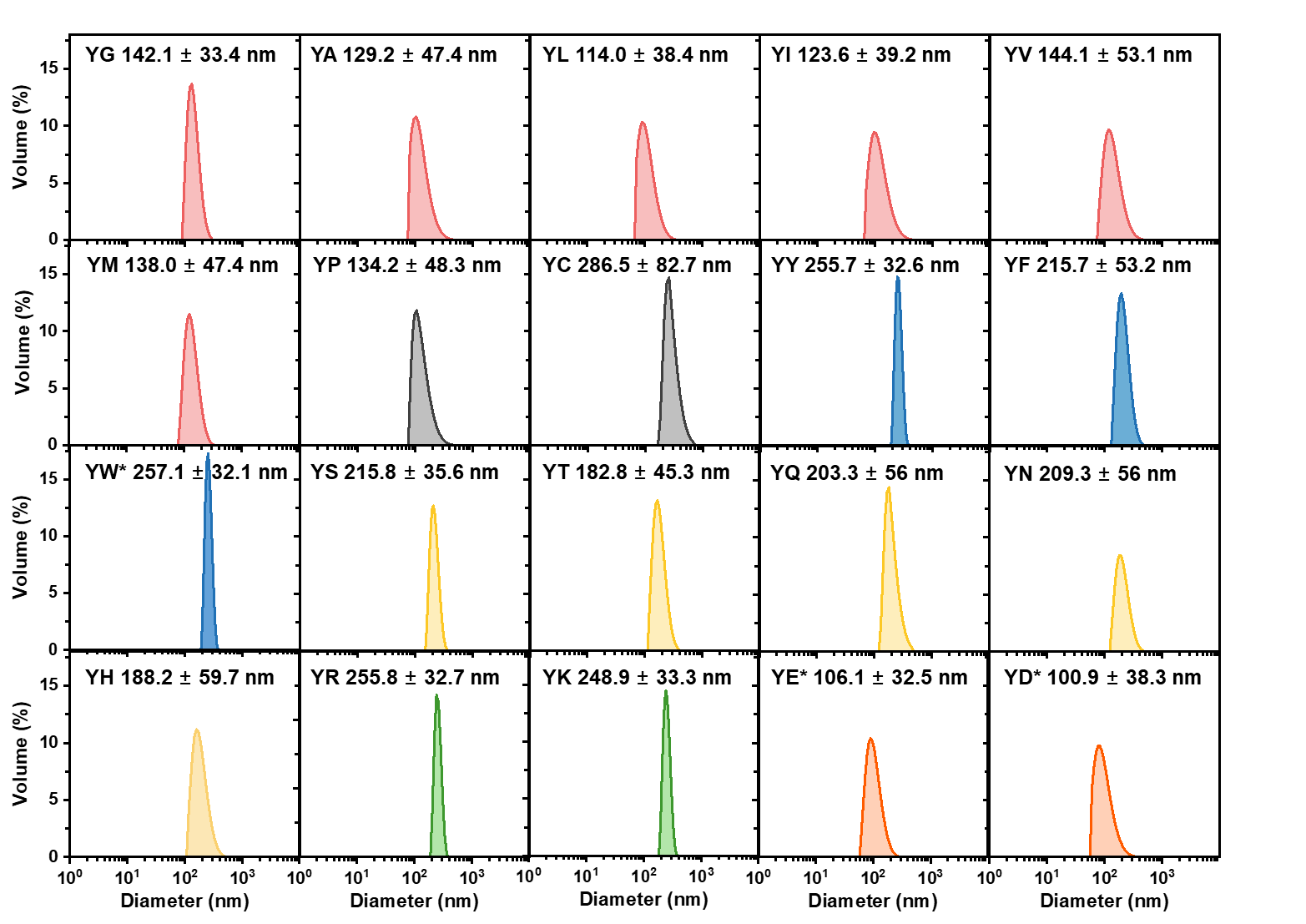


**Figure S2.** The DLS size distributions of all 20 YX peptide nanoparticles assembled at a peptide concentration of 0.5 mg mL^−1^. * indicates the use of 80:20 (v/v) DMF:pH 10 buffer solution for YW and a peptide concentration of 2.0 mg mL^−1^ for YE and YD.


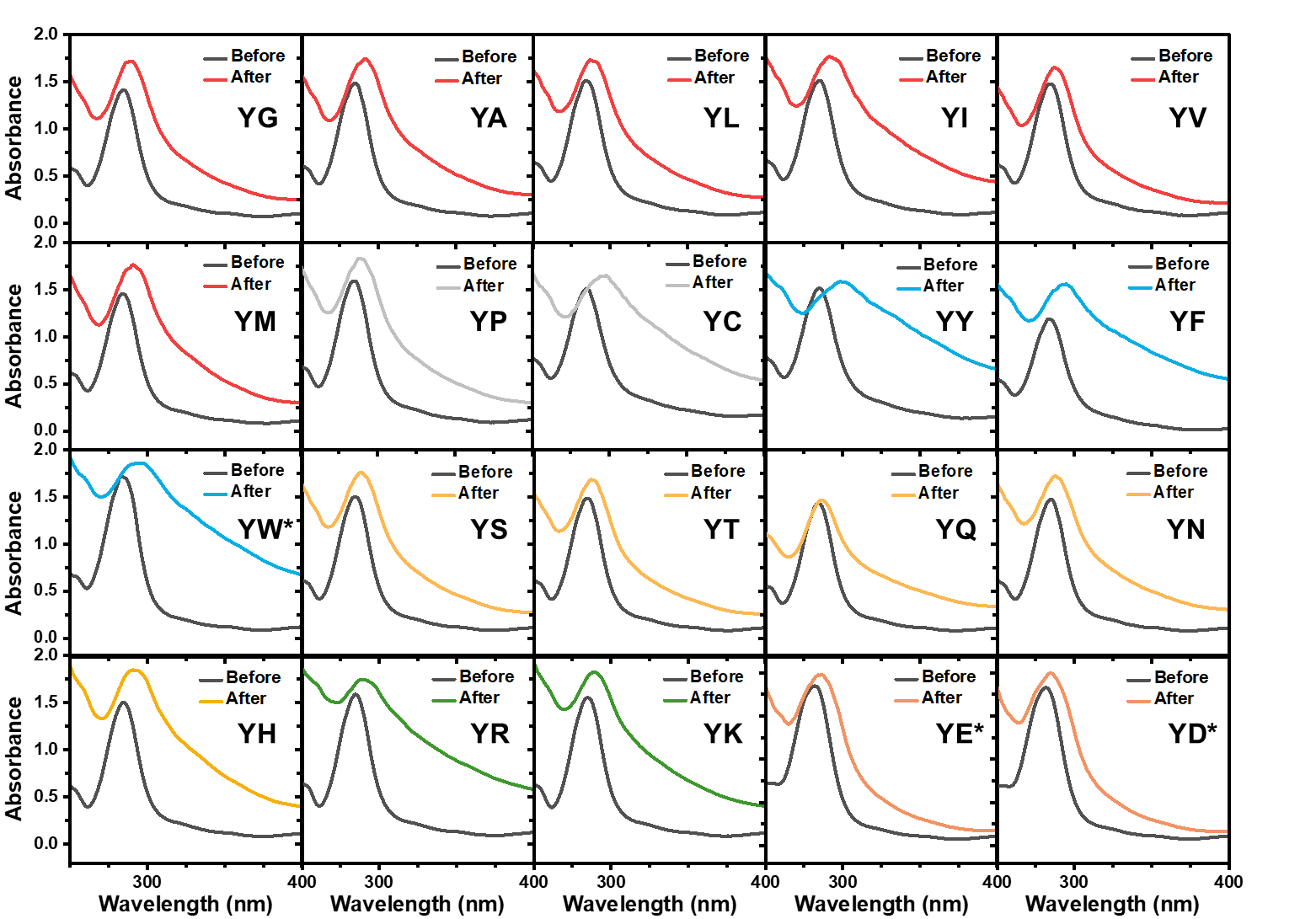


**Figure S3.** The UV-vis spectra of the 20 YX peptide mixtures recorded before (black) and after (colored) covalent assembly for 10 min. The spectral changes indicate the formation of covalent crosslinks and the structural evolution of the peptide assemblies during the tyrosine crosslinking-induced assembly.


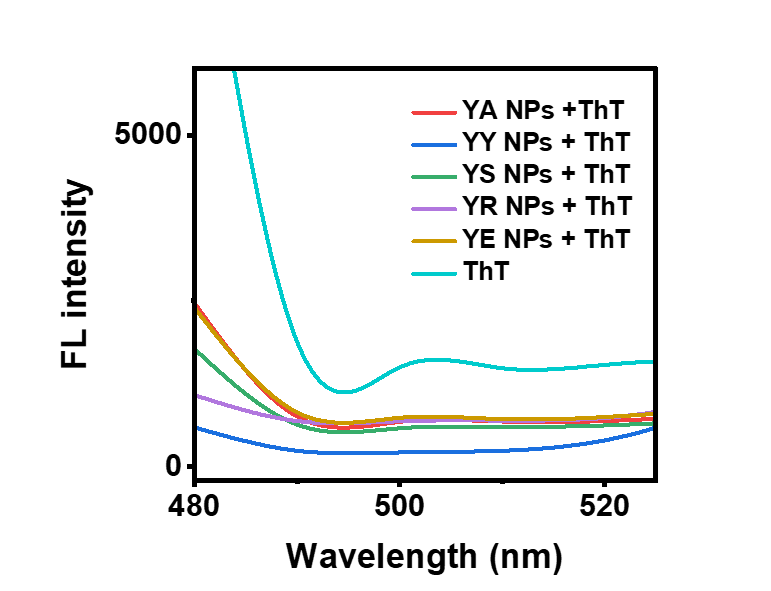


**Figure S4.** The fluorescence spectra of five representative YX nanoparticles stained with ThT and a control (only ThT), indicating the absence of β-sheet conformation within the assemblies.


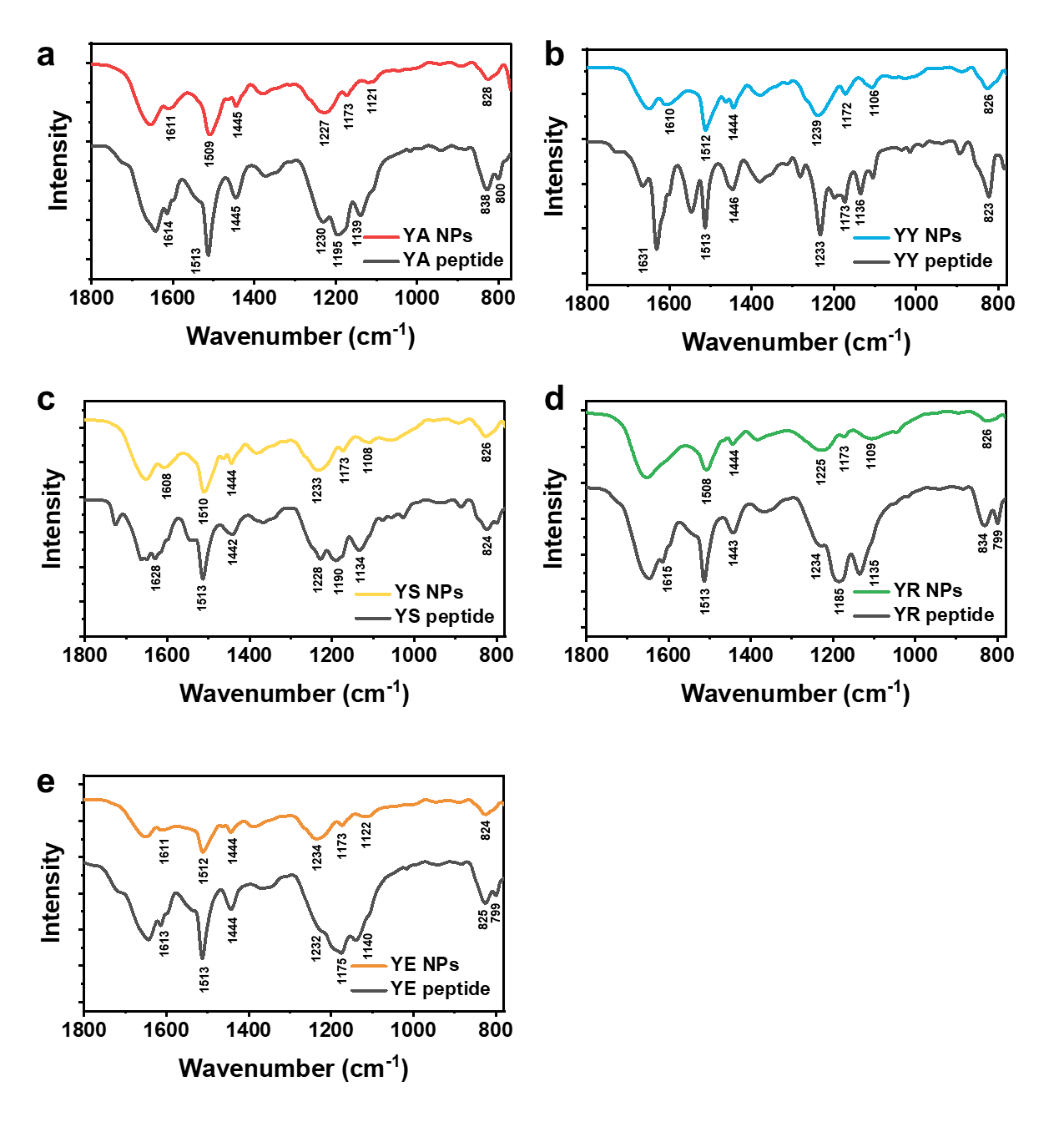


**Figure S5.** The FT-IR spectra of the YX peptide monomers (black) and YX peptide nanoparticles (colored): (a) YA, (b) YY, (c) YS, (d) YR, and (e) YE.

**
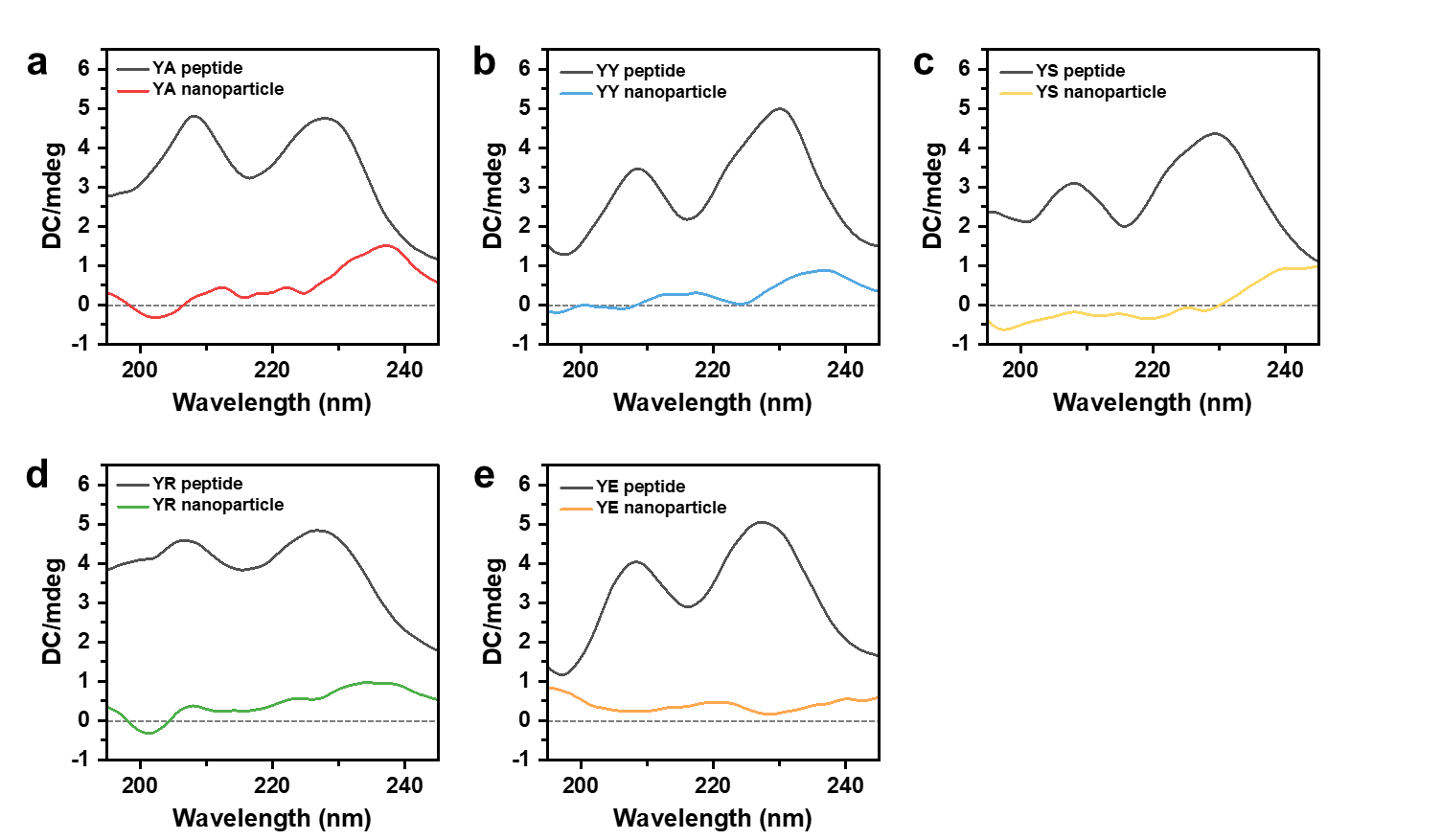
**

**Figure S6.** The CD spectra of the YX peptide monomers (black) and YX peptide nanoparticles (colored): (a) YA, (b) YY, (c) YS, (d) YR, and (e) YE.


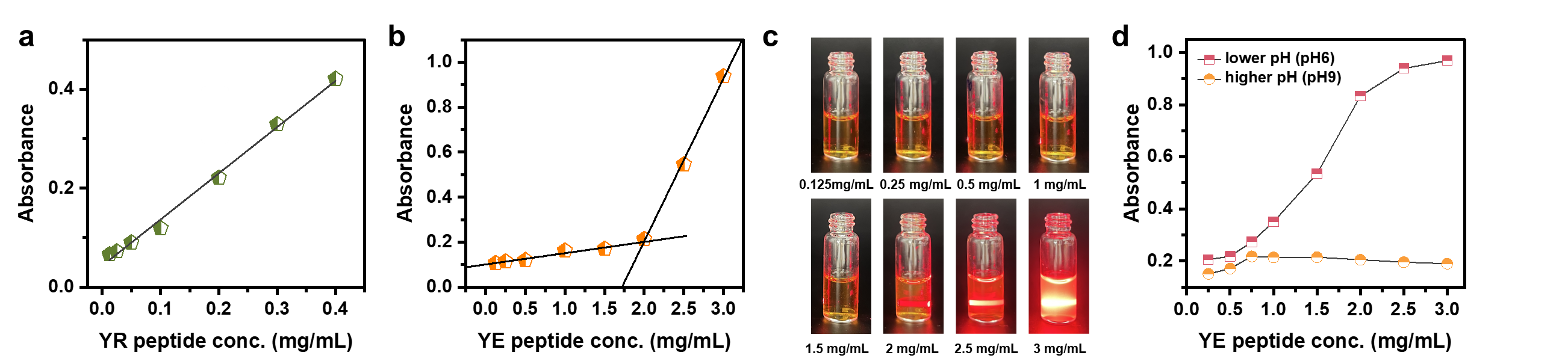


**Figure S7.** (a) The absorbance of the YR peptide solution after assembly as a function of peptide concentration, showing the linear relationship between concentration and absorbance. (b) The plot of absorbance intensity as a function of YE peptide concentration at pH ~ 8, for use in determining the critical aggregation concentration. (c) Photographic images of the YE peptide solutions at various concentrations, demonstrating the Tyndall effect due to the presence of peptide nanoparticles above 2 mg mL^−1^. (d) The plots of absorbance of the YE peptide solution as a function of concentration after assembly under the conditions of pH 6 and pH 9.


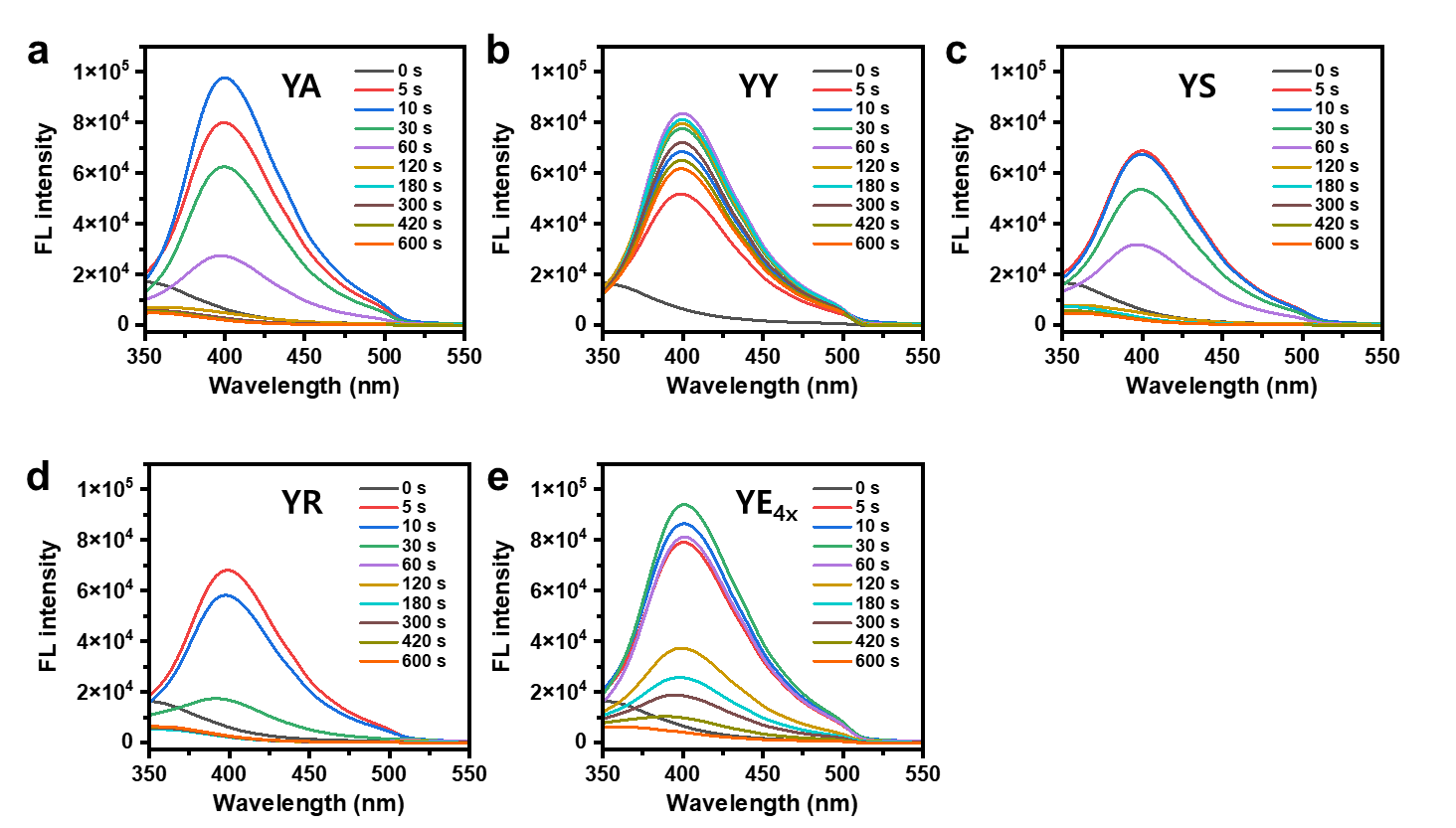


**Figure S8.** The time-dependent fluorescence emission spectra of representative YX peptides at 315 nm excitation: (a) YA, (b) YY, (c) YS, (d) YR, and (e) YE at 4x concentration.


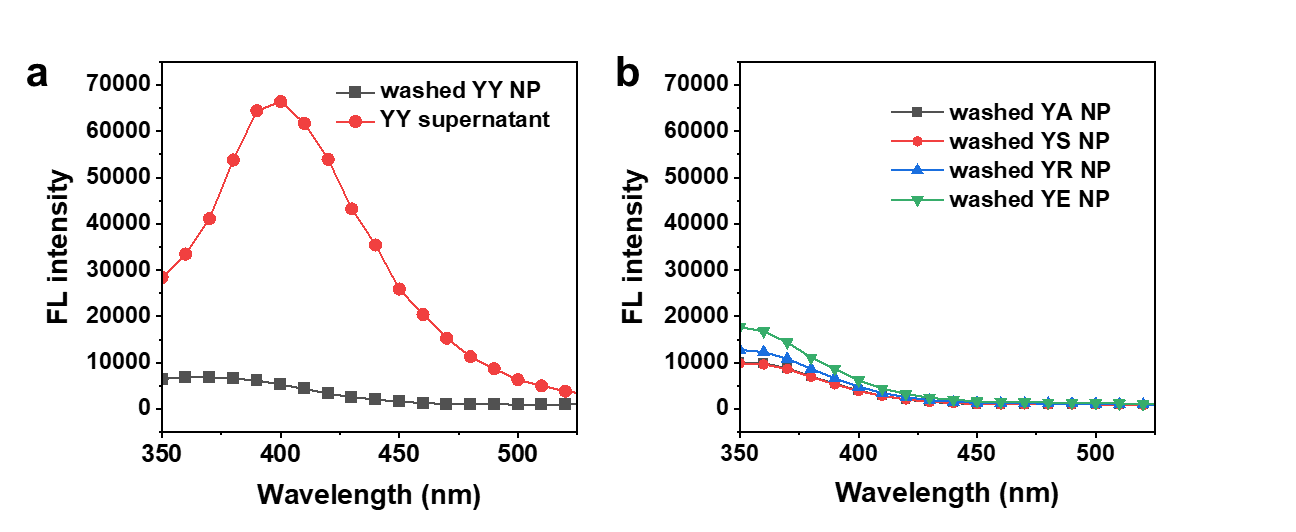


**Figure S9.** (a) The fluorescence emission spectra of the purified YY nanoparticles (gray) and the corresponding supernatant (red) at 315 nm excitation. The fluorescence detected in the YY supernatant indicates the presence of loosely associated or unassembled peptide species in the solution, while fluorescence quenching is observed in the nanoparticles due to aggregation-caused quenching. (b) The fluorescence emission spectra of the purified YA, YS, YR, and YE, nanoparticles excited at 315 nm, showing aggregation-caused quenching.


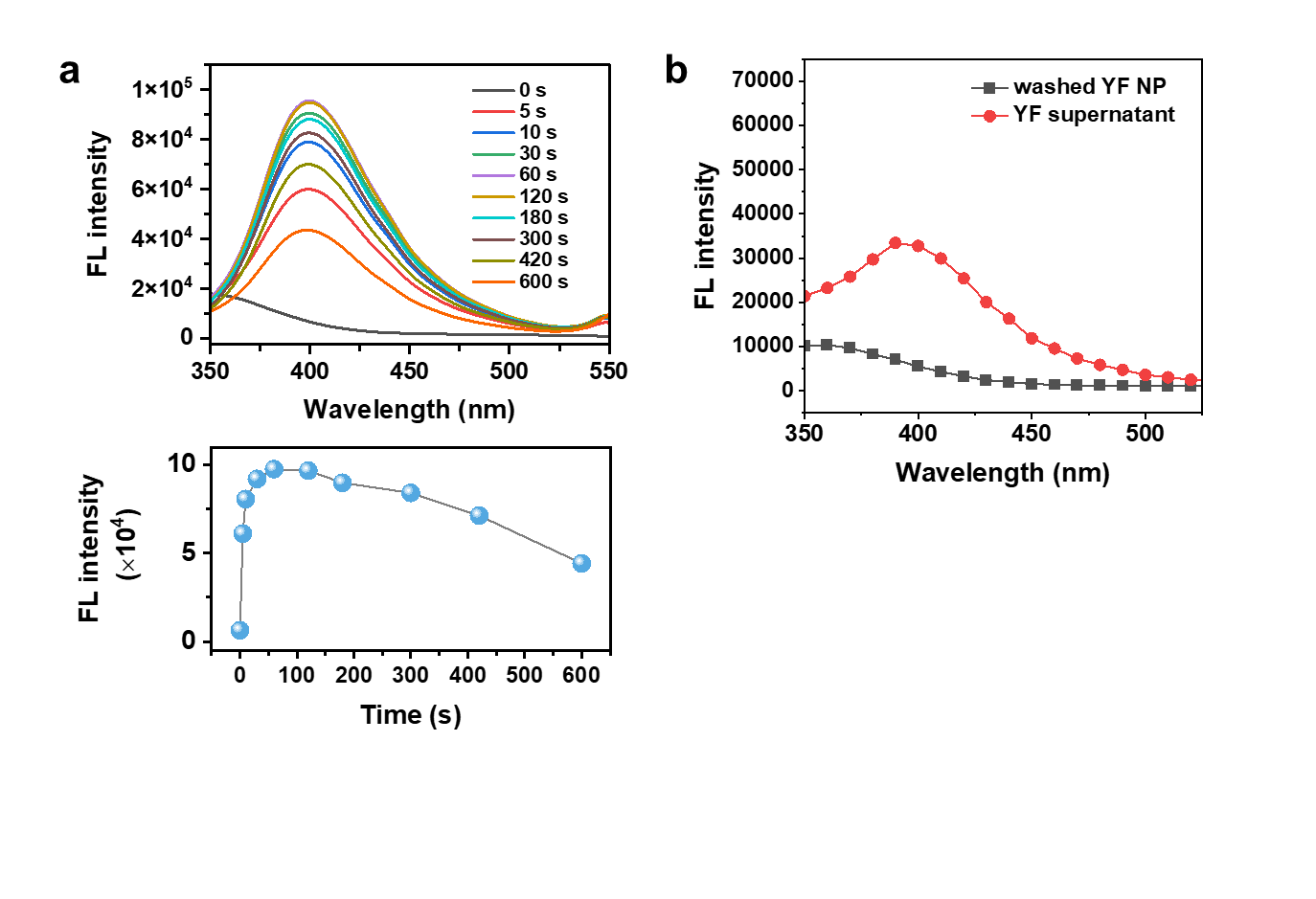


**Figure S10.** (a) The time-dependent fluorescence emission spectra (top) and profile (bottom) of the YF peptide solution at 400 nm (315 nm excitation). (b) The fluorescence emission spectra (315 nm excitation) for the washed YF nanoparticles (gray) and the corresponding supernatant phase (red) after centrifugation. The YF exhibits a low aggregation-caused quenching efficiency of 61.43%, with the remaining fluorescence intensity being attributed to non-participating crosslinked monomers in the supernatant, similar to that observed in the YY peptide.


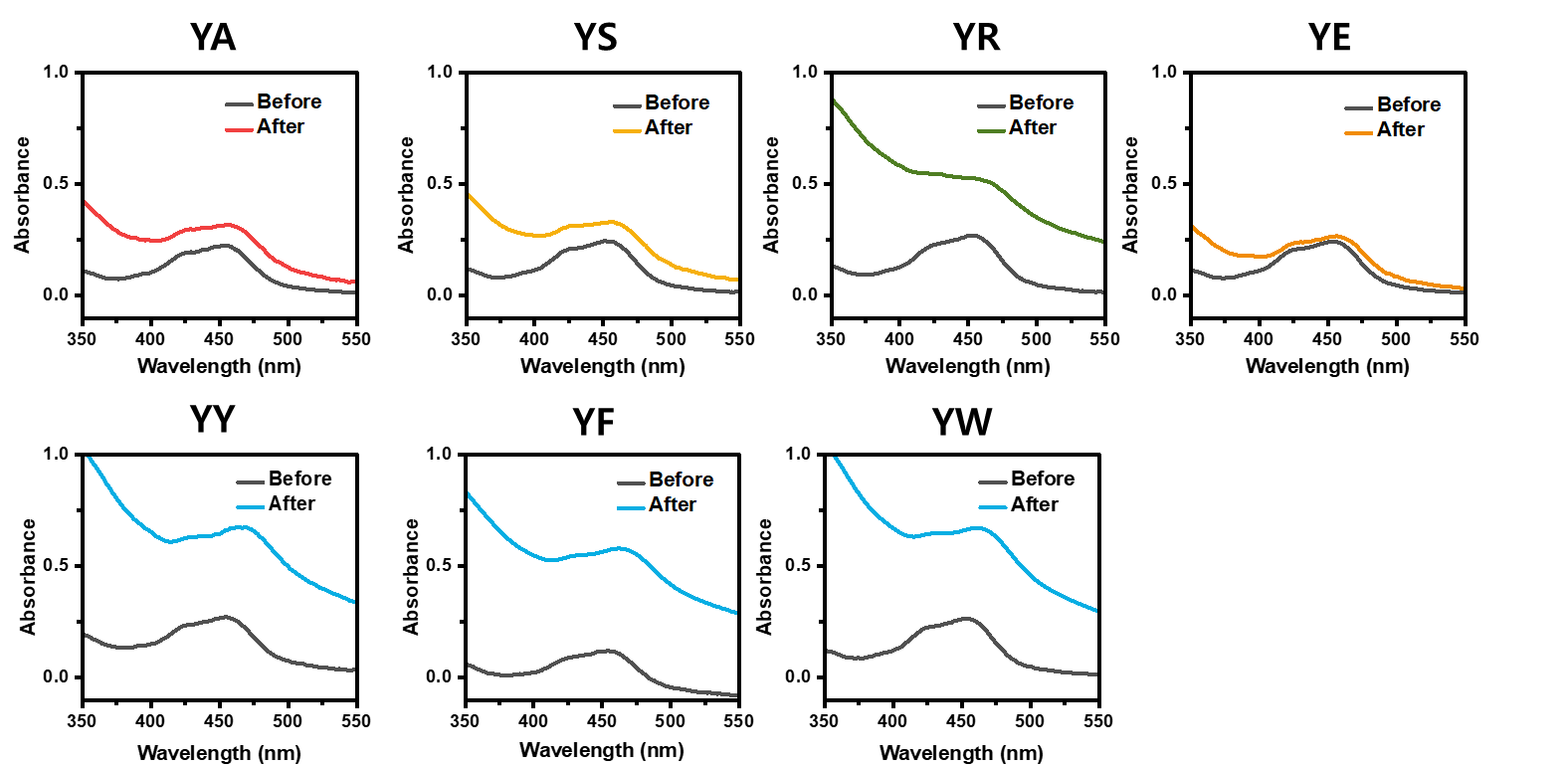


**Figure S11.** The comparative UV-vis spectra of the YX reaction mixtures before (gray) and after (colored) the covalent peptide assembly. Here, the absorption band at ~454 nm due to the metal-to-ligand charge transfer (MLCT) of the Ru complex exhibits a red-shift after nanoparticle formation across all YX peptides. This shift is attributed to the intensification of intermolecular interactions and electron-donating interactions between the peptide and the pyridyl ligand within the tightly aggregated nanoparticles.^[1]^ Notably, the peptides containing aromatic amino acids, such as YY, YF, and YW demonstrate a more pronounced red-shift, reflecting stronger electronic interactions facilitated by their aromatic side chains.


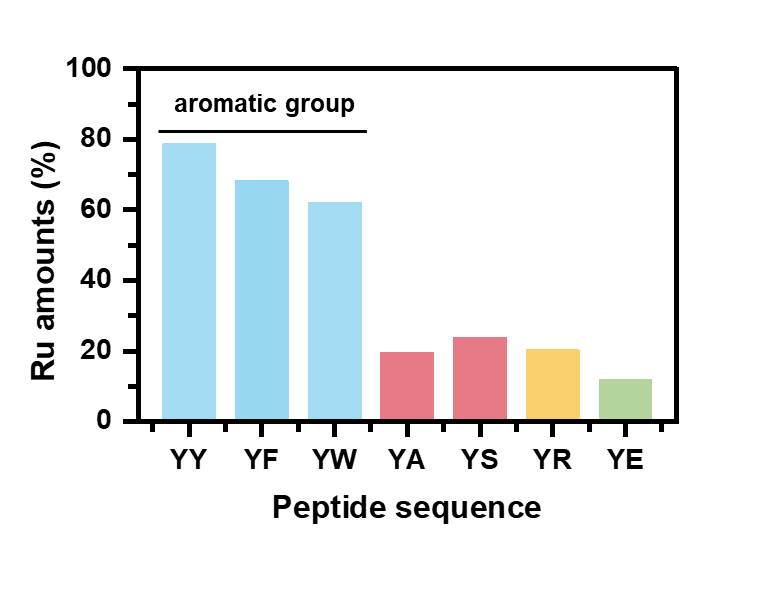


**Figure S12.** A comparison of the amounts of Ru confined within YX peptide nanoparticles, expressed as a percentage of its initial concentration. The assembly process utilized 0.17 mM of Ru(bpy)_3_Cl_2_. After assembly and three cycles of centrifugation, the Ru contents in the YA, YS, YR, and YE nanoparticles were reduced to approximately 10–25% of their initial values, corresponding to concentrations below 50 μM. For the YY nanoparticles, which exhibited the highest Ru uptake (79%, 134 μM), disassembly reduced the Ru content to below 10% in the resulting hollow nanoparticles, thereby ensuring very low residual levels.


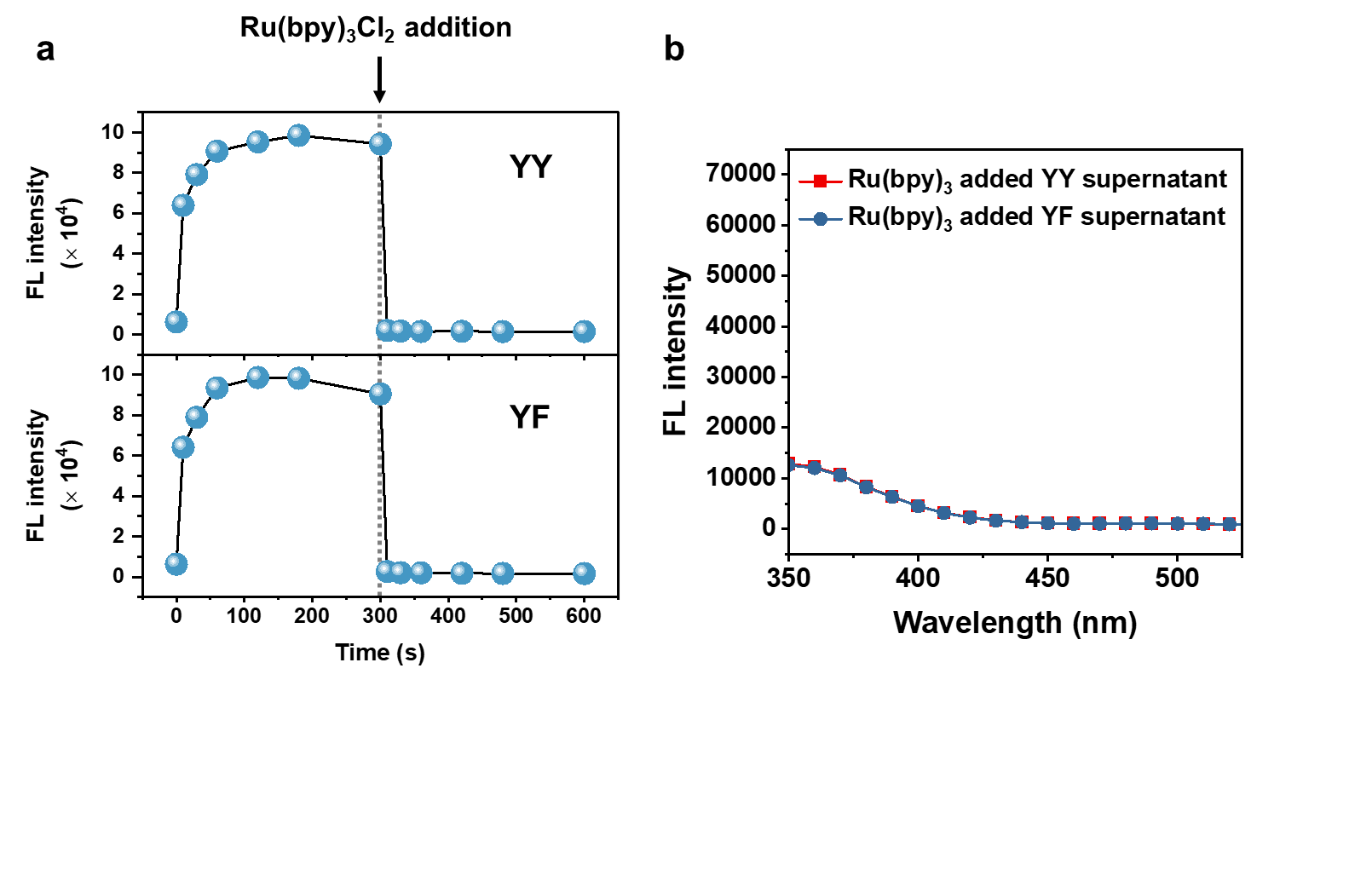


**Figure S13.** (a) The fluorescence intensity plots of the YY (top) and YF (bottom) reaction mixtures, showing complete quenching upon the addition of supplementary Ru(bpy)_3_Cl_2_ (equivalent to the initial amount) at the reaction midpoint. (b) The fluorescence spectra of the fully quenched supernatants obtained after the reaction, confirming the absence of loosely associated or unassembled peptide species in the solution.


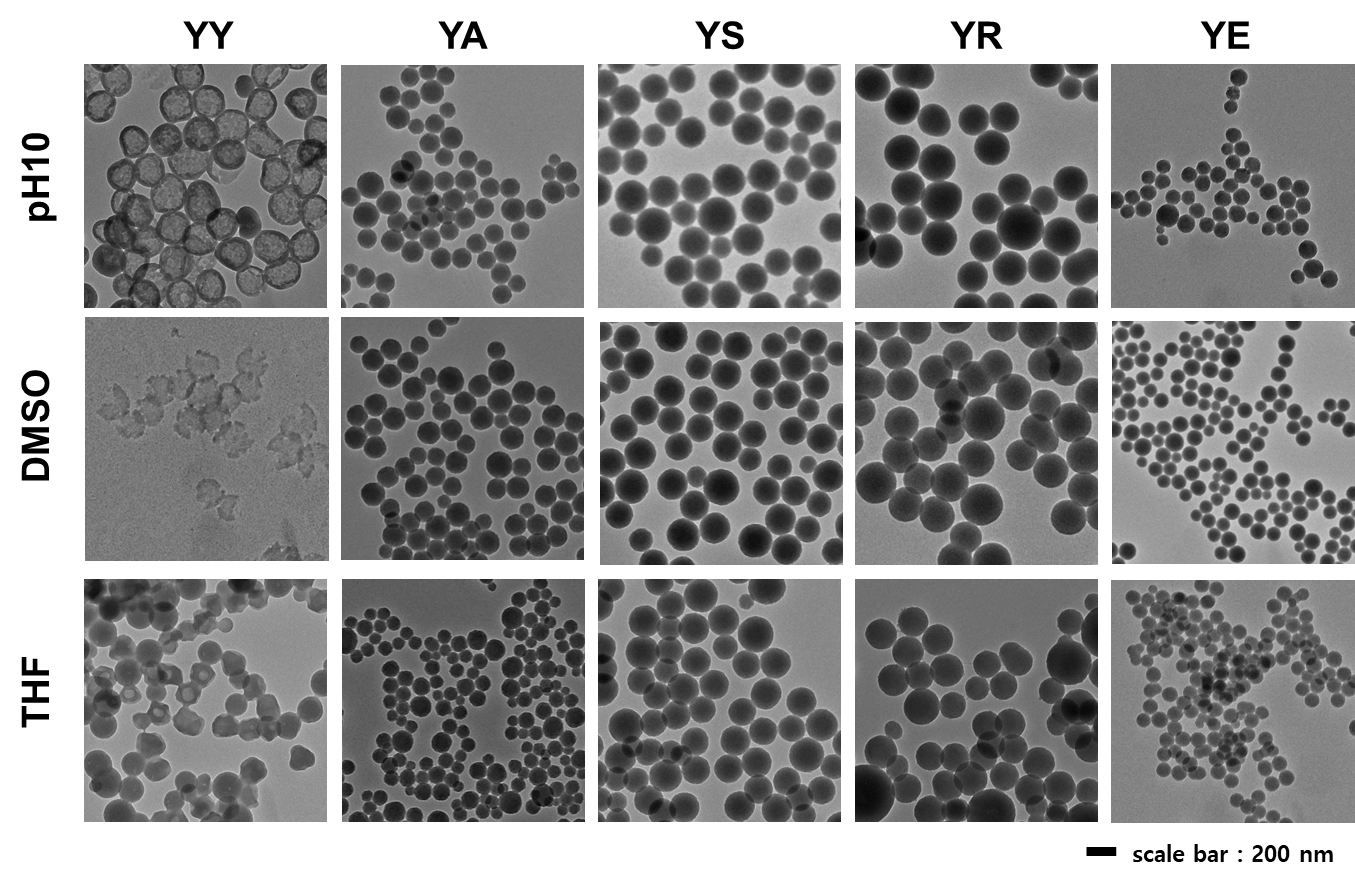


**Figure S14.** The TEM images of YY, YA, YS, YR, and YE nanoparticles after 10 min of incubation in a pH 10 buffer (top row), 20:80 (v/v) DMSO:water (middle row), and 20:80 (v/v) THF:water (bottom row).


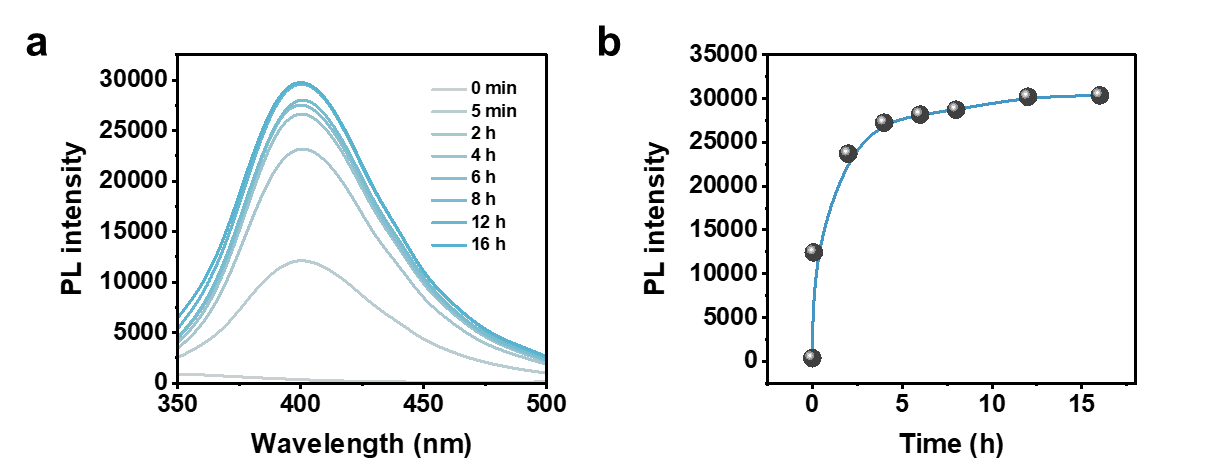


**Figure S15.** The time-dependent fluorescence emission spectra (a) and corresponding intensity profile at 400 nm (b) demonstrating the gradual recovery of blue fluorescence. This behavior reflects the release of aggregated peptide compartments from the less crosslinked network within the nanoparticles.


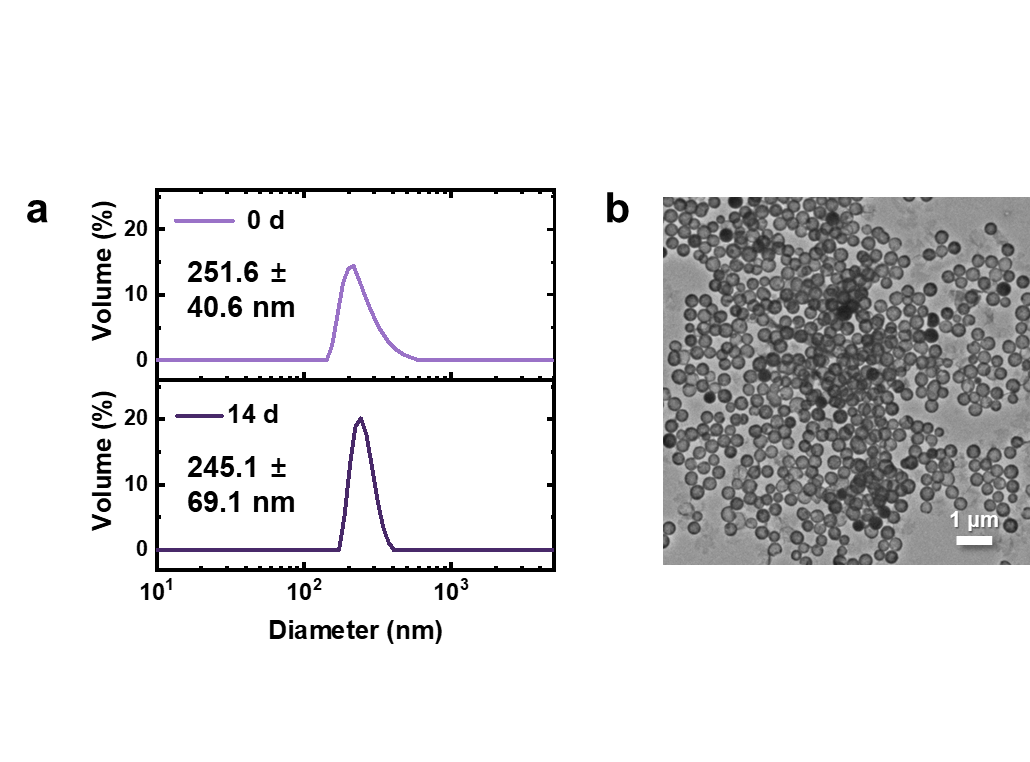


**Figure S16.** The long-term storage stability of the YY hollow nanoparticles: (a) the size distributions of YY hollow nanoparticles immediately after assembly (top) and after 14 days in aqueous storage (bottom), demonstrating no size variation and stable dispersity. (b) a TEM image of the YY hollow nanoparticles after 14 days of storage, revealing the preserved structural integrity and morphology, with no significant degradation or collapse of the hollow structure.


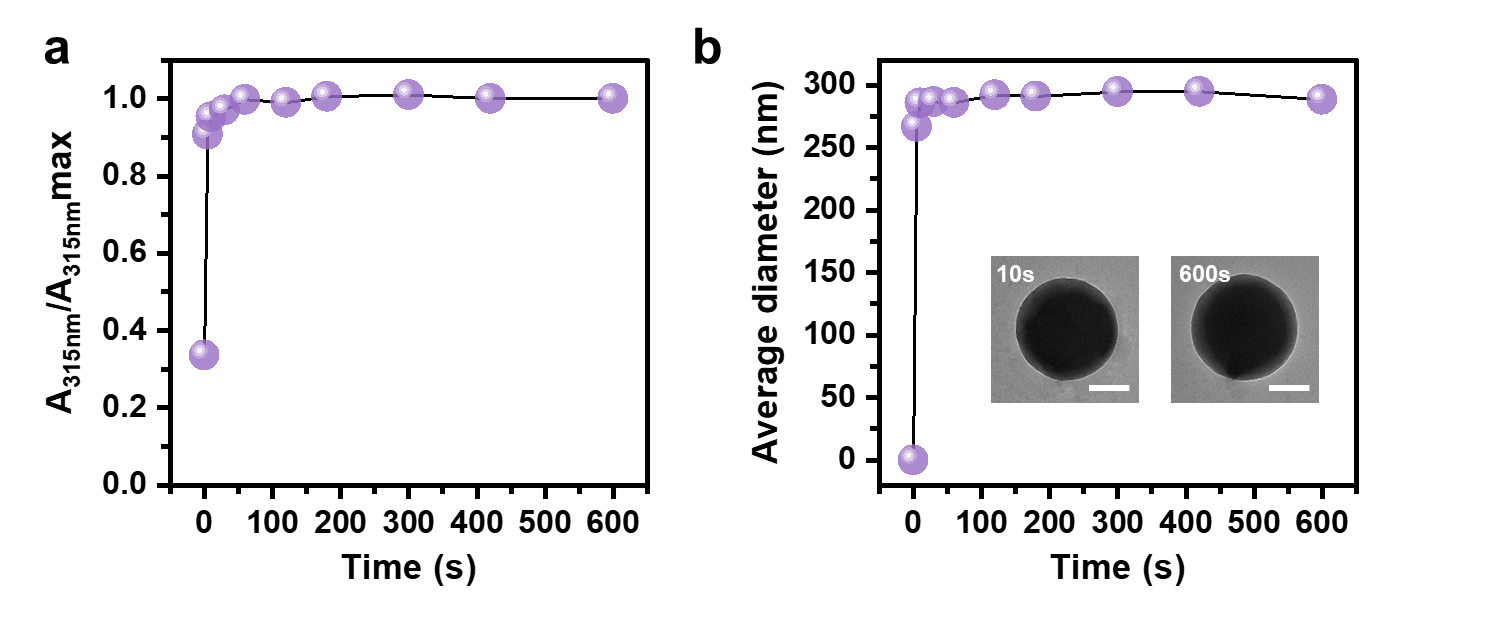


**Figure S17.** (a) The time-dependent normalized UV-vis absorption profile of the YC peptide at 315 nm. (b) The change in average diameter of the YC peptide nanoparticles over time, and the corresponding TEM images (inset; scale bar = 100 nm).


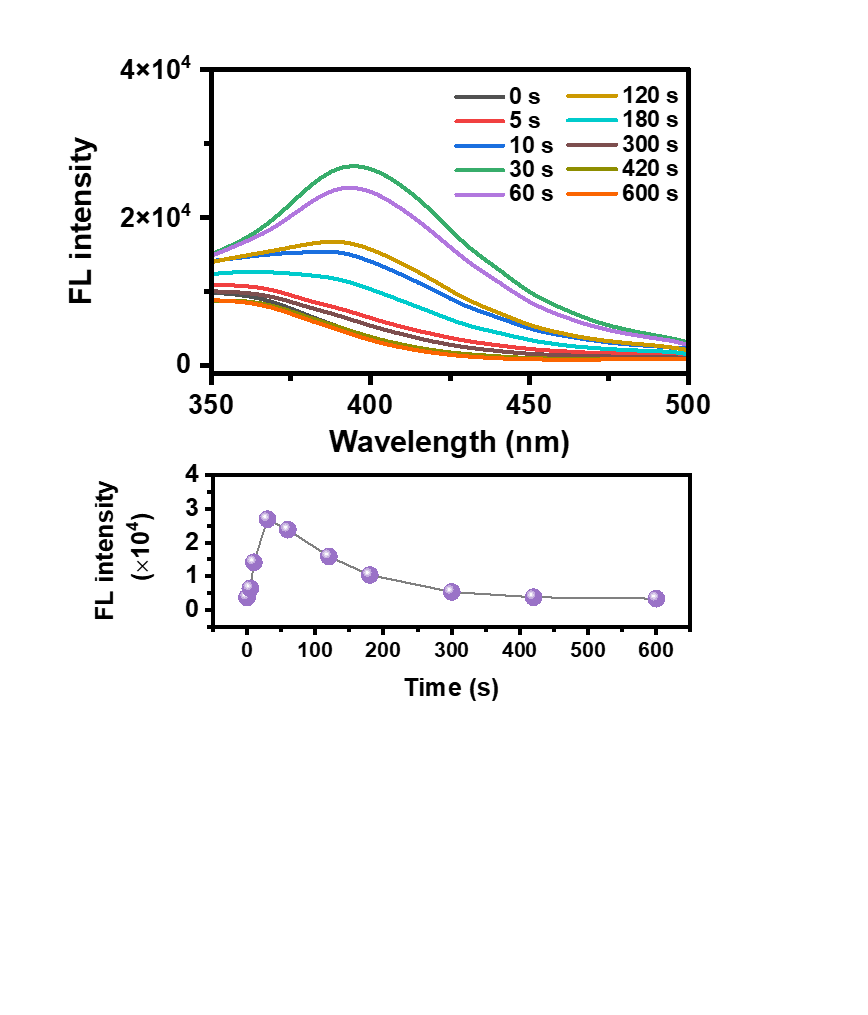


**Figure S18.** The time-dependent fluorescence spectra of the YC peptide (top) and the corresponding intensity profile at 400 nm (excitation at 315 nm) (bottom).


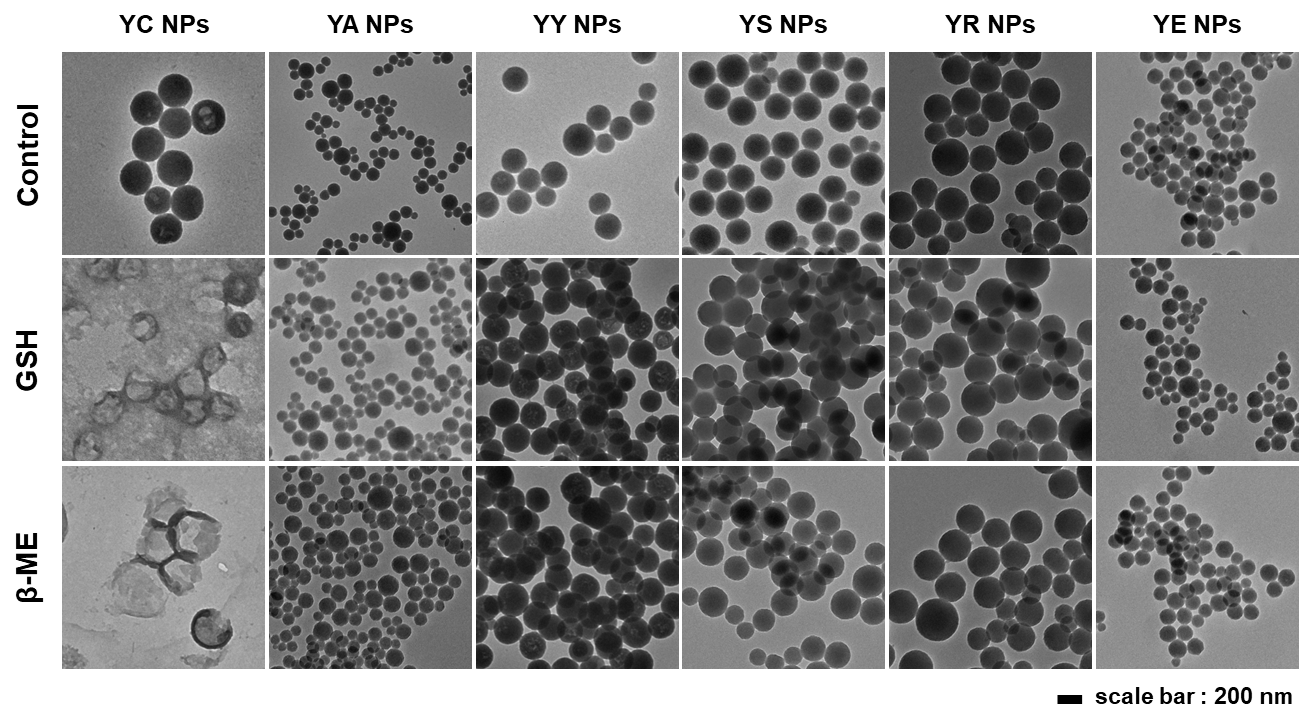


**Figure S19.** The TEM images of the YC and YX (X = A, Y, S, R, E) nanoparticles before (top row) and after treatment 0.5 mM GSH (middle row) and 1% β-ME (bottom row) for 10 min. The YC nanoparticles are significantly disassembled in the presence of disulfide bond-disrupting agents, while the other YX peptides remain unchanged. This demonstrates the stimuli-responsive disassembly of the YC peptide, which is attributed to the presence of disulfide bonds in its structure. (Note: the YY nanoparticles show slight leaching of their interiors, with preservation of the overall structural integrity. This minor leaching occurred during subsequent washing steps due to their lower crosslinking density.)

**
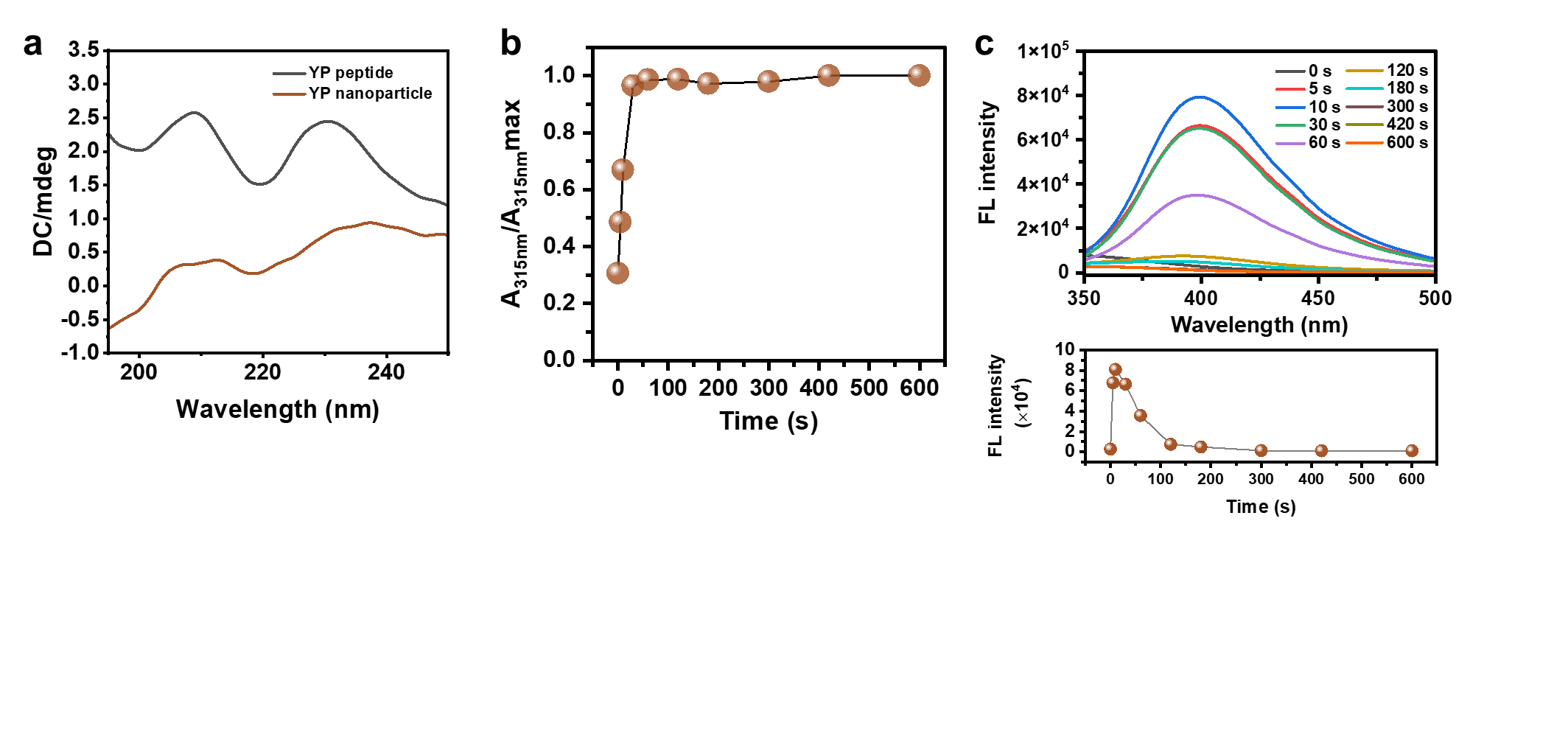
**

**Figure S20.** (a) The CD spectra of the YP peptide monomer (black) and YP peptide nanoparticle (brown). (b) Time-dependent normalized UV–vis absorption profiles at 315 nm. (c) The fluorescence spectra of the YP peptide at 400 nm.

**
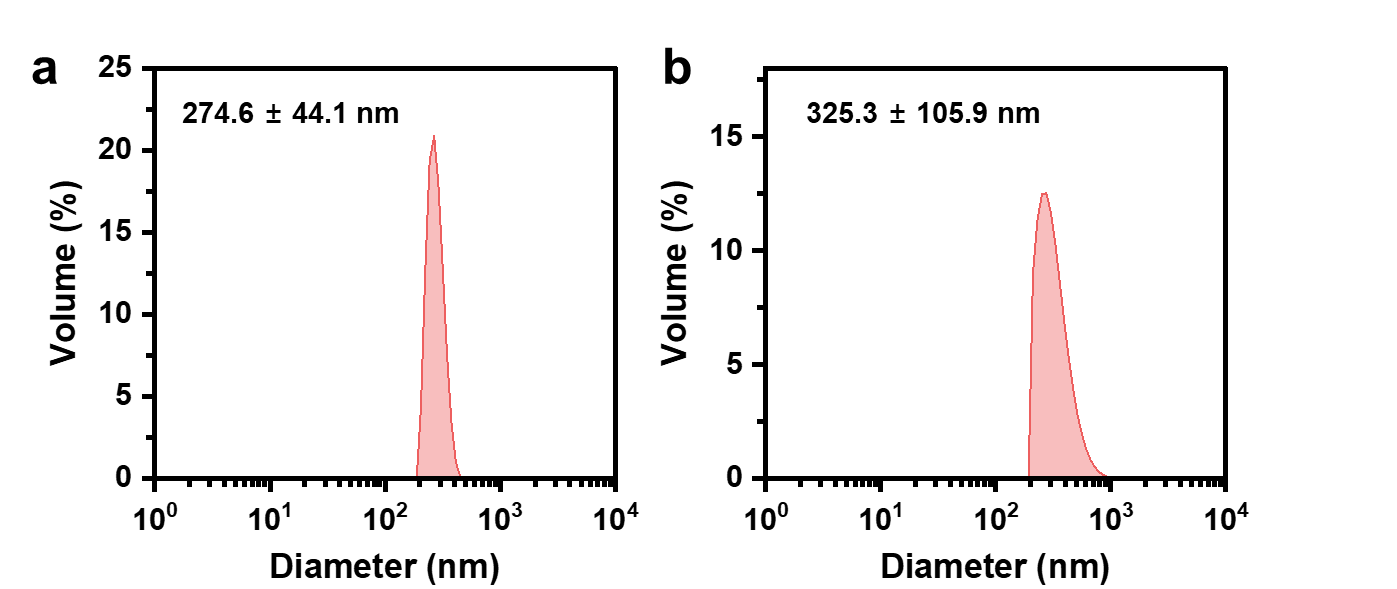
**

**Figure S21.** The size distributions of (a) the biotin-YG nanoparticles and (b) the RhB-YH nanoparticles.


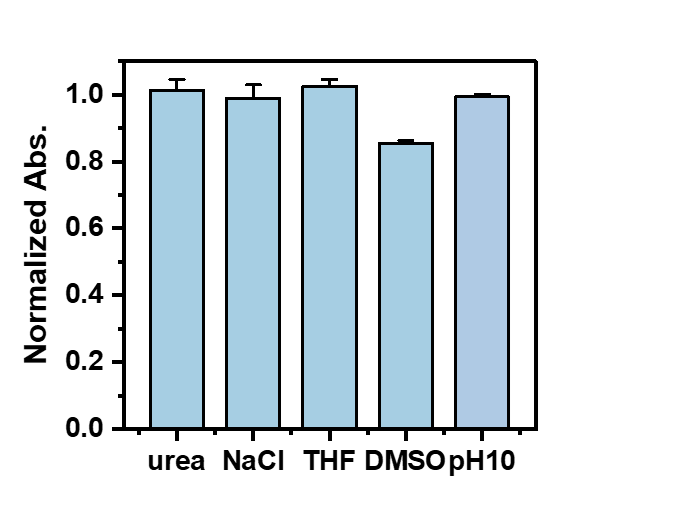


**Figure S22.** The normalized absorbances of the YG nanoparticles after treatment with 100 mM urea, 100 mM NaCl, 20:80 THF:water (v/v), 20:80 DMSO:water (v/v), and pH 10 buffer.


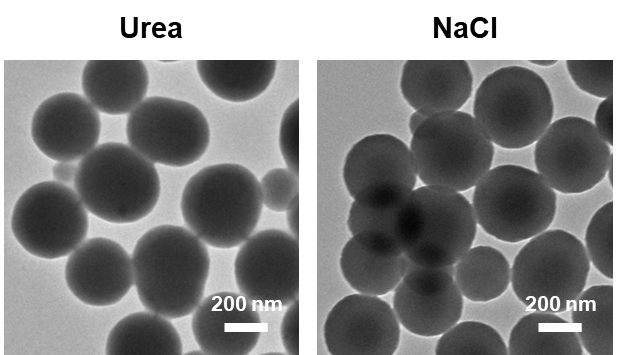


**Figure S23.** The TEM images of the biotin-YG nanoparticles after treatment with 100 mM urea (left) and 100 mM NaCl (right) for 10 min. Despite the decrease in absorbance, the nanoparticles maintain their overall structure, thereby suggesting that the disruption of noncovalent interactions via urea or NaCl treatment is insufficient to induce a significant morphological transformation.


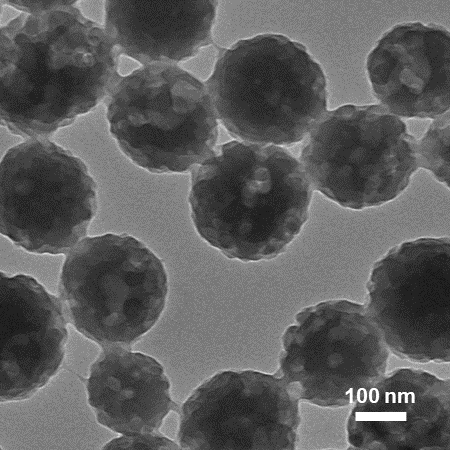


**Figure S24.** TEM image of the dimpled biotin-YG nanoparticles after 30 days of storage, revealing the preserved structural integrity and morphology.


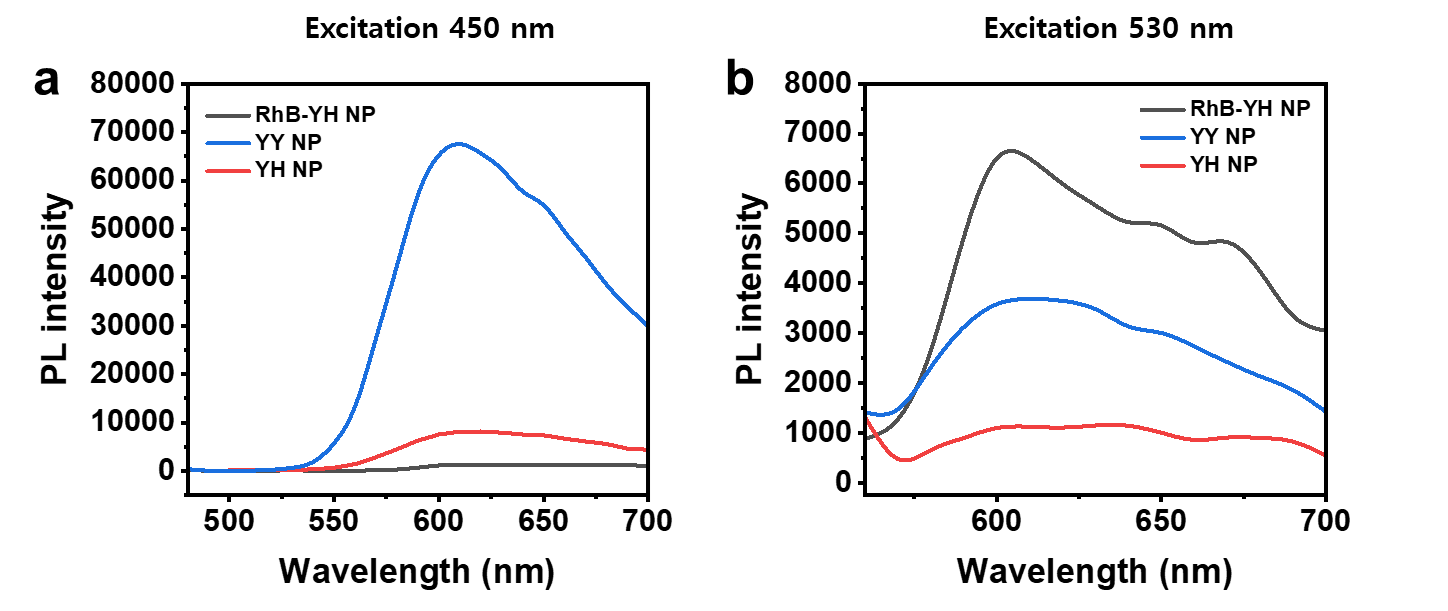


**Figure S25.** The PL spectra of the RhB-YH (gray), YY (blue), and YH (red) nanoparticles with excitation at (a) 450 nm and (b) 530 nm. The YH nanoparticles did not exhibit any distinct optical properties.


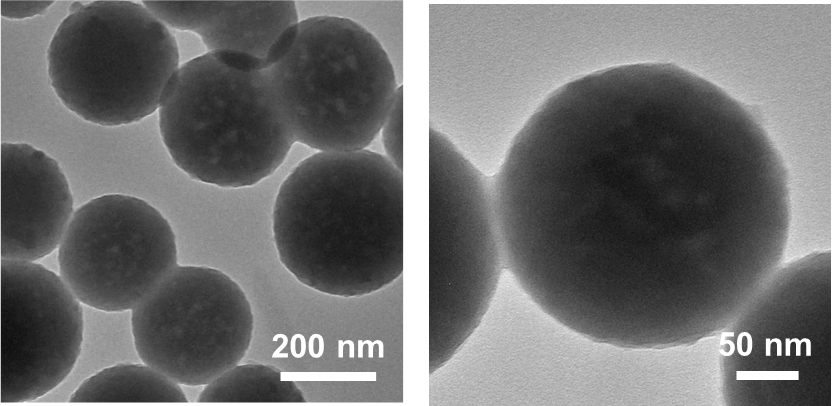


**Figure S26.** The TEM images of the partially disassembled RhB-YH nanoparticles after the addition of DMSO (50:50 v/v relative to RhB-YH nanoparticle aqueous solution), followed by incubation for 30 s.


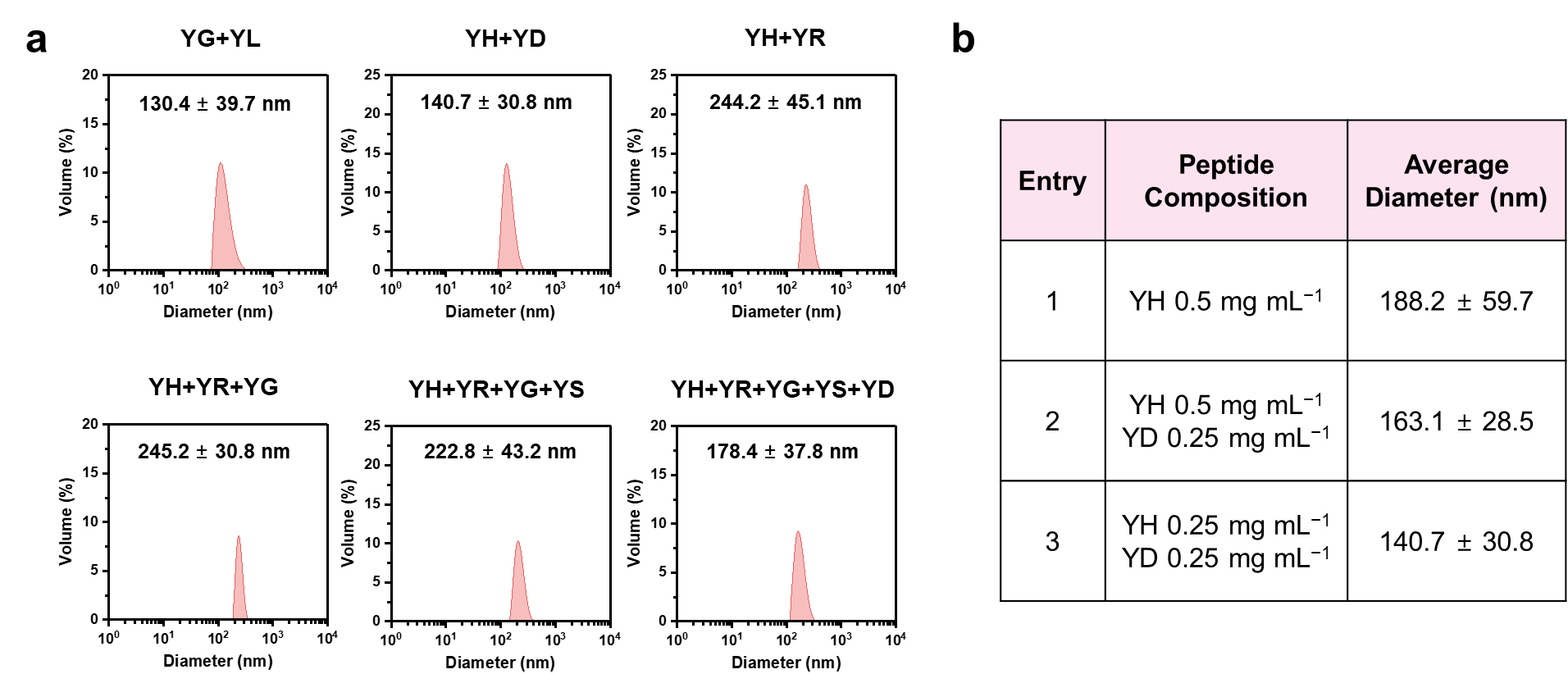


**Figure S27.** (a) The size distributions of the co-assembled YX nanoparticles incorporating binary (YG+YL, YH+YD, and YH+YR), ternary (YH+YR+YG), quaternary (YH+YR+YG+YS), and quinary (YH+YR+YG+YS+YD) peptide modules. (b) The average sizes and peptide compositions of assemblies formed by the YH and YD peptides, showing the size reduction effect of YD.


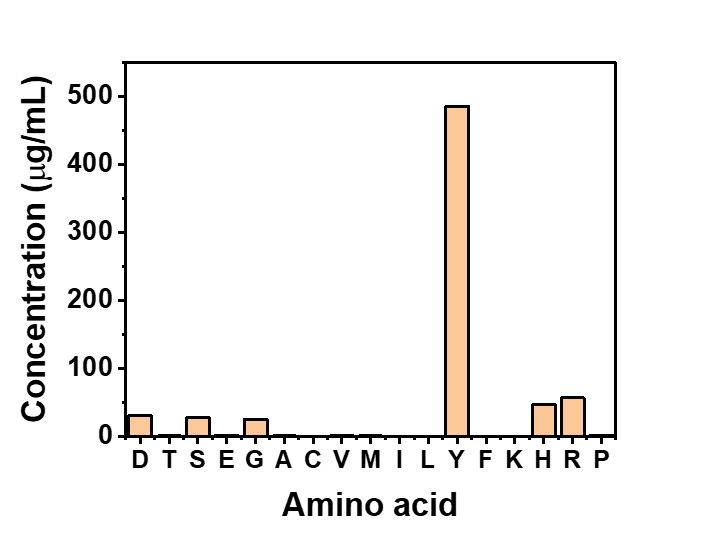


**Figure S28.** The amino acid analysis of the YX nanoparticles formed by multiple modular assembly incorporating YH, YR, YG, YS, and YD peptide modules, confirming the successful incorporation of the different peptide modules into the assembly.


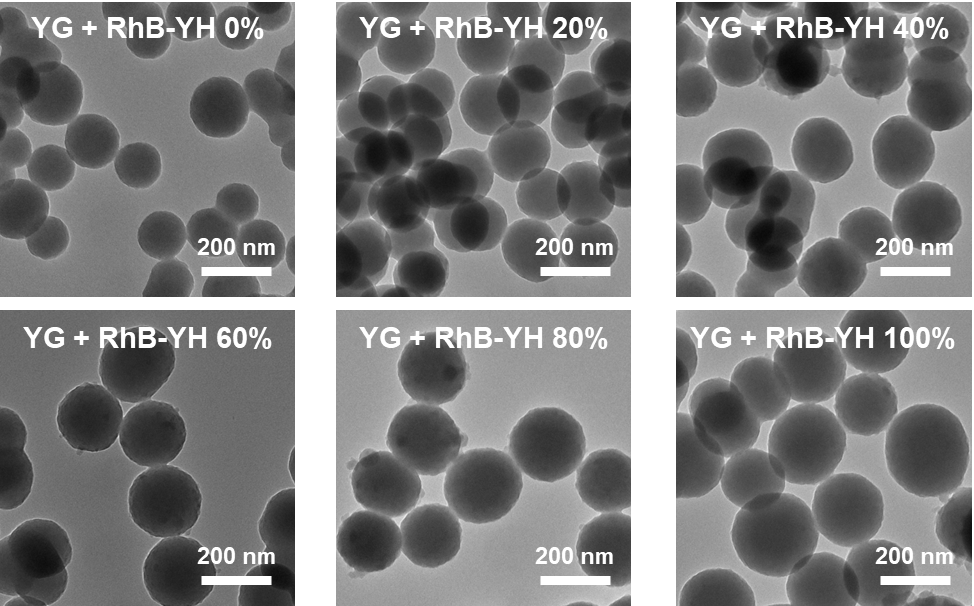


**Figure S29.** The TEM images of the YG@RhB-YH nanoparticles co-assembled using various amounts of additional RhB-YH (0%, 20%, 40%, 60%, 80%, and 100%) relative to a fixed concentration of YG (0.5 mg mL^−1^).


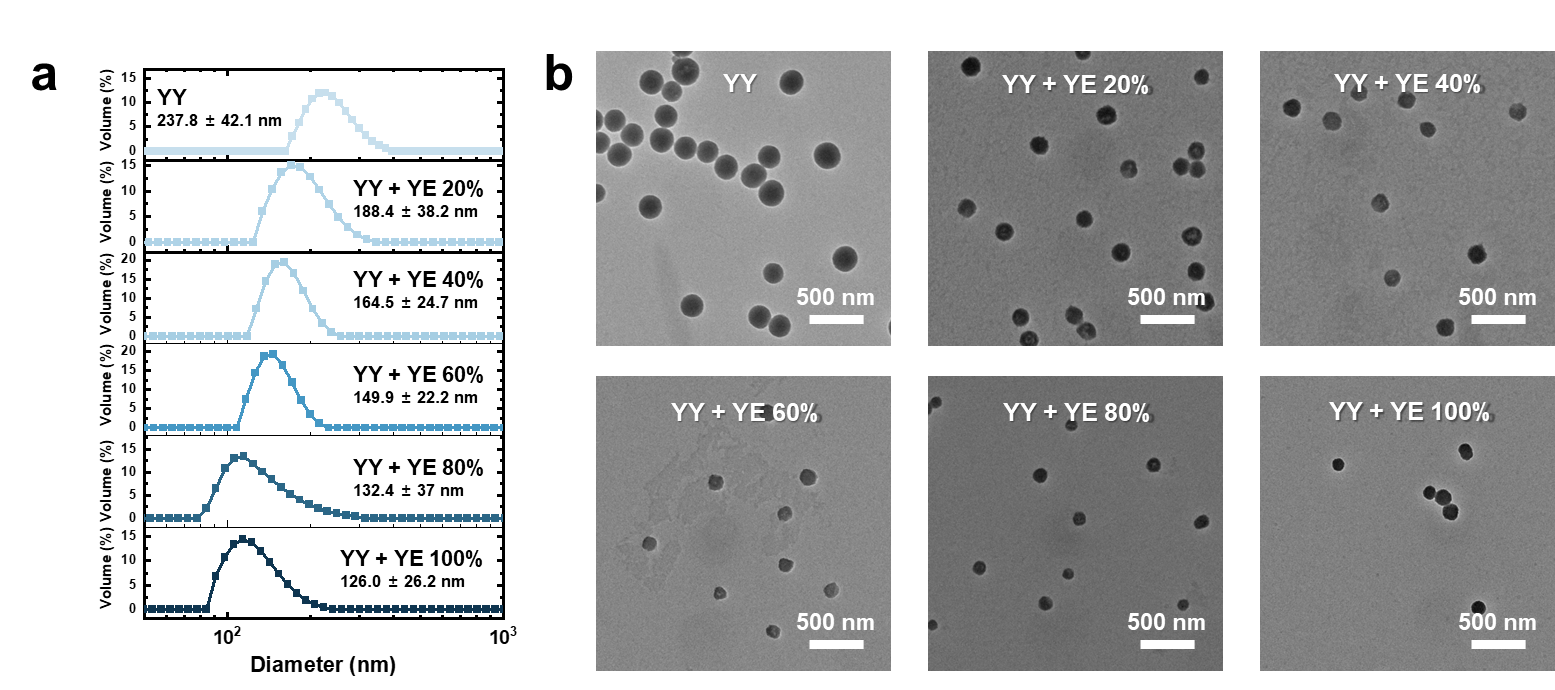


**Figure S30.** The size distributions (a) and TEM images (b) of a series of co-assembled YY@YE peptide nanoparticles with various concentrations of additional YE (0–100%) relative to a fixed concentration of YY (0.5 mg mL^−1^). These data provide insights into how the addition of YE peptide influences the size of the co-assembled YY@YE nanoparticles, acting as a growth frustrating factor and potentially altering the nanoparticle size by interfering with the growth dynamics.

**
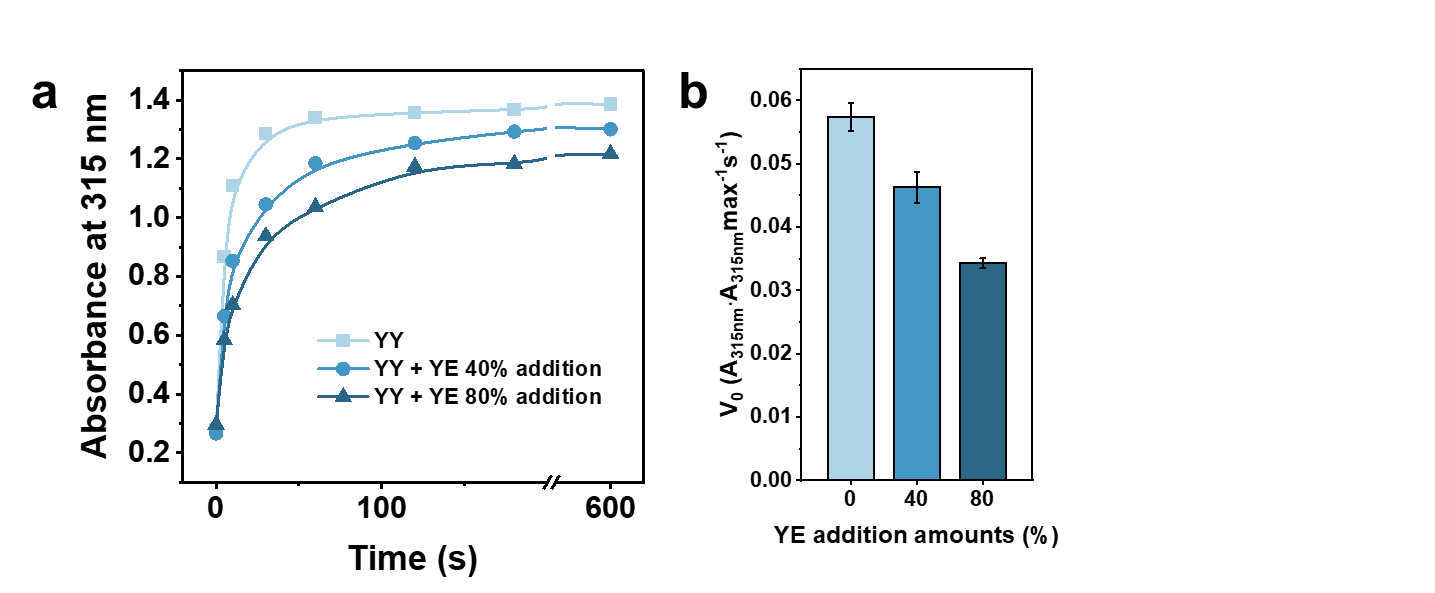
**

**Figure S31.** The time-dependent UV-vis absorbance at 315 nm (a) and initial formation rate (b) of YE@YY nanoparticles assembled with various concentrations of additional YE (0%, 40%, and 80%) relative to a fixed concentration of YY (0.5 mg mL^−1^). These data provide insights into the kinetics of nanoparticle formation, illustrating how the addition of various amounts of YE peptide influences the assembly process, including the rate of formation and changes in absorbance over time.


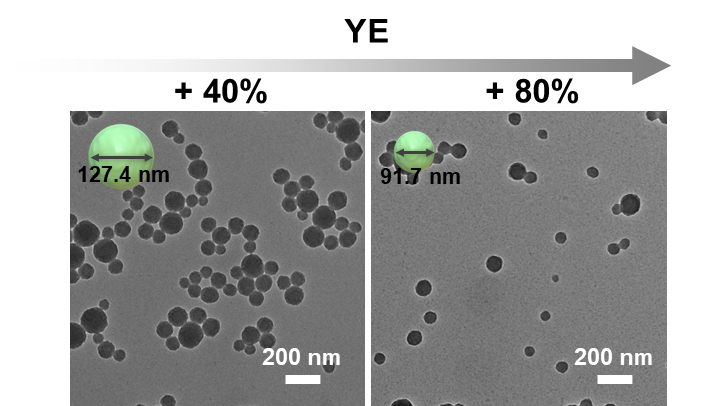


**Figure S32.** The TEM images of the co-assembled YA@YE nanoparticles with the addition of 40% (left) and 80% (right) YE peptide to a fixed concentration of YA peptide (0.5 mg mL^−1^). Here, the nanoparticle size is seen to decrease as the amount of YE is increased.


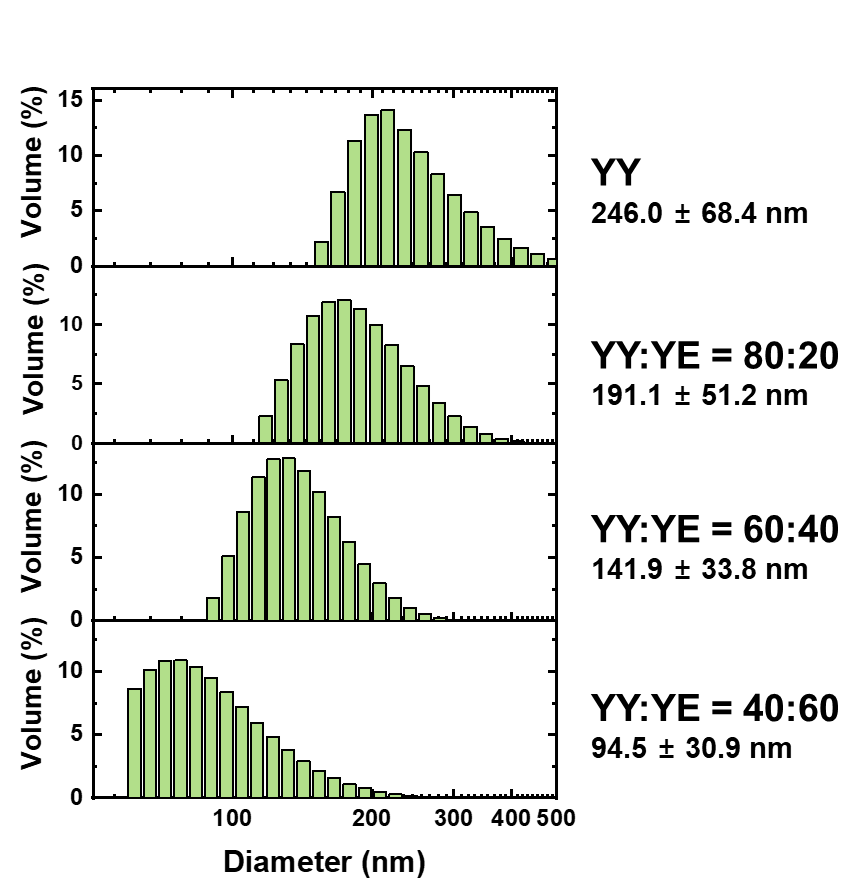


**Figure S33.** The size distributions of the co-assembled YY@YE nanoparticles with various compositional ratios of YY:YE peptides (100% YY and 80:20, 60:40, and 40:60 YY:YE (w/w)) with a final concentration of 0.5 mg mL^−1^.


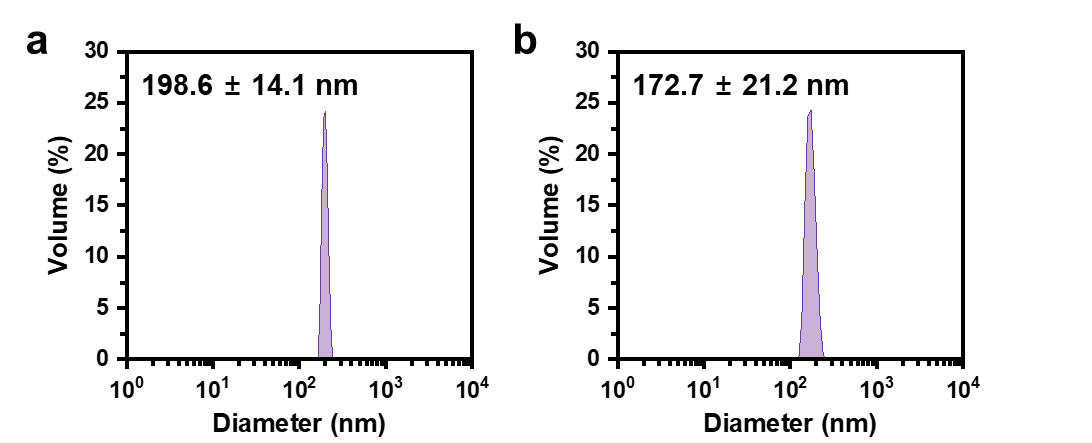


**Figure S34.** The size distributions of the co-assembled biotin-YG@YG (a) and biotin-YG@YY (b) nanoparticles.

**Table S1**. A summary of the key characteristics of the series of 20 YX peptides.

| **Amino acid**  **(X)** | **Properties** | **Average diameter (nm)** | **Zeta potential (pH7, mV)** | **UV-vis. peak shift (nm)** | **ΔAbs_315nm_** | **Yield (%)** |
| --- | --- | --- | --- | --- | --- | --- |
| **Glycine (G)** | **nonpolar**  **non-charged** | 142.1 ± 33.4 | -24.04 ± 0.64 | 285→290 (Δ5) | 0.59 | 90.81 ± 0.42 |
| **Alanine (A)** |  | 129.2 ± 47.4 | -26.2 ± 3.91 | 285→291 (Δ6) | 0.705 | 87.84 ± 0.26 |
| **Lysine (K)** |  | 114.0 ± 38.4 | -23.46 ± 1.39 | 284→289 (Δ5) | 0.583 | 87.74 ± 0.09 |
| **Isoleucine (I)** |  | 123.6 ± 39.2 | -25.29 ± 2.2 | 285→291 (Δ6) | 0.916 | 90.74 ± 0.22 |
| **Valine (V)** |  | 144.1 ± 53.1 | -21.15 ± 2.23 | 285→288 (Δ3) | 0.438 | 84.38 ± 0.16 |
| **Methionine (M)** |  | 138.0 ± 37.6 | -30.23 ± 1.14 | 284→291 (Δ7) | 0.755 | 95.74 ± 0.17 |
| **Proline (P)** | **special case** | 134.2 ± 48.3 | -24.04 ± 0.64 | 285→290 (Δ5) | 0.59 | 90.81 ± 0.42 |
| **Cysteine (C)** |  | 286.5 ± 82.7 | -26.2 ± 3.91 | 285→291 (Δ6) | 0.705 | 87.84 ± 0.26 |
| **Tyrosine (Y)** | **nonpolar**  **non-charged**  **aromatic** | 255.7 ± 32.6 | -27.43 ± 1.7 | 285→298 (Δ13) | 1.045 | 70.86 ± 0.54 |
| **Phenylalanine (F)** |  | 215.7 ± 53.2 | -28.69 ± 1.36 | 284→295 (Δ11) | 0.983 | 68.2 ± 0.83 |
| **Tryptophan (W)*** |  | 257.1 ± 32.1 | -25.8 ± 1.15 | 283→295 (Δ12) | 1.278 | 67.3 ±0.46 |
| **Serine (S)** | **polar**  **non-charged** | 215.8 ± 35.6 | -33.7 ± 2.8 | 285→288 (Δ3) | 0.608 | 81.81 ± 0.28 |
| **Threonine (T)** |  | 182.8 ± 45.3 | -20.9 ± 8.4 | 285→287 (Δ2) | 0.522 | 80.05 ± 0.43 |
| **Glutamine (Q)** |  | 203.3 ± 56 | -32.6 ± 0.3 | 285→287 (Δ2) | 0.534 | 90.01 ± 0.22 |
| **Asparagine (N)** |  | 209.3 ± 56 | -26.7 ± 2.1 | 285→287 (Δ2) | 0.621 | 85.34 ± 0.2 |
| **Histidine (H)** |  | 188.2 ± 59.7 | -17.1 ± 1.1 | 285→292 (Δ7) | 0.984 | 94.87 ± 0.06 |
| **Arginine (R)** | **polar**  **(+) charged**  **base** | 255.8 ± 32.7 | -20.6 ± 0.7 | 285→290 (Δ5) | 1.028 | 96.69 ± 0.12 |
| **Lysine (K)** |  | 248.9 ± 33.3 | -28.9 ± 1.2 | 285→289 (Δ4) | 0.861 | 96.66 ± 0.5 |
| **Glutamic acid (E)*** | **polar**  **(-) charged**  **acid** | 106.1 ± 32.5 | -37.16 ± 1.1 | 282→286 (Δ4) | 0.482 | 33.75 ± 0.51 |
| **Aspartic acid (D)*** |  | 100.9 ± 38.3 | -36.59 ± 1.8 | 282→285 (Δ3) | 0.463 | 33.86 ± 1.27 |

*2.0 mg mL^−1^ for YD and YE, 2:8 DMF:pH 10 buffer (v/v) for YW.

**Table S2.** The frequencies of the FTIR absorption markers for five representative YX peptide sequences.^[2–6]^

| **Group assignments** | **YA** | **YY** | **YS** | **YR** | **YE** |
| --- | --- | --- | --- | --- | --- |
| **Aromatic ring C–C stretching vibration** | **1614 cm^−1^** (decrease, shift) | **1631 cm^−1^** (decrease, shift) | **1628 cm^−1^** (decrease, shift) | **1615 cm^−1^** (disappear) | **1613 cm^−1^** (decrease, shift) |
| **Aromatic C=C stretching vibration** | **1513 cm^−1^** (decrease, shift) | **1513 cm^−1^** (decrease, shift) | **1513 cm^−1^** (decrease, shift) | **1513 cm^−1^** (decrease, shift) | **1513 cm^−1^** (decrease, shift) |
| **C–H bending vibration** | **1445 cm^−1^** (decrease) | **1446 cm^−1^** (decrease, shift) | **1442 cm^−1^** (decrease, shift) | **1443 cm^−1^** (decrease) | **1444 cm^−1^** (decrease) |
| **Phenolic C–OH bending vibration** | **1230 cm^−1^** (decrease, shift) | **1233 cm^−1^** (decrease, shift) | **1228 cm^−1^** (decrease, shift) | **1234 cm^−1^** (decrease, shift) | **1232 cm^−1^** (decrease, shift) |
| **Phenolic C–OH stretching vibration** | **1195 cm^−1^** (decrease, shift) | **1173 cm^−1^** (decrease, shift) | **1190 cm^−1^** (decrease, shift) | **1185 cm^−1^** (decrease, shift) | **1175 cm^−1^** (decrease, shift) |
| **Aromatic ring C–H bending** | **1139 cm^−1^** (decrease, shift) | **1136 cm^−1^** (decrease, shift) | **1134 cm^−1^** (decrease, shift) | **1135 cm^−1^** (decrease, shift) | **1140 cm^−1^** (decrease, shift) |
| **C–H bending, disubstituted** | **838, 800 cm^−1^** (merge) | **823 cm^−1^** (shift) | **824 cm^−1^** (shift) | **834, 799 cm^−1^** (decrease, merge) | **825, 799 cm^−1^** (decrease, merge) |

**Table S3.** A summary of the assembly compositions and conditions for the multicomponent modular co-assembly of YX peptides. In each case, the total reaction volume = 1 mL.

| **Entry** | | **Peptide**  (2.5 mg mL**^−1^**) | **Co-assembly** | **Ru(bpy)_3_Cl_2_** | **APS** | **Water** |
| --- | --- | --- | --- | --- | --- | --- |
| **Figure 5b and c** | **YG@RhB-YH**  (RhB-YH addition amount) | **YG**  DI water  **RhB-YH**  DMSO | **YG**  200 μL  **RhB-YH**  0–200 μL | 0.85 mM,  200 μL | 60 mM,  100 μL | 300–500 μL |
| **Figure 5d** | **YY@YE**  (YE addition amount) | **YY**  pH 10 buffer  **YE**  pH 10 buffer | **YY**  200 μL  **YE**  0–200 μL |  |  |  |
| **Figure 5e** | **YY@YE**  (YY:YE ratio) |  | **YY** (X%)  + **YE** (100-X%)  200 μL  X = 40–100 |  | 10 mM,  600 μL | – |
| **Figure 5f** | **Biotin-YG@YG** | **Biotin-YG**  20:80 DMF:pH 10 buffer (v/v)  **YG**  pH 10 buffer | **Biotin-YG**  100 μL  **YG**  100 μL |  |  |  |
| **Figure 5f** | **Biotin-YG@YY** | **Biotin-YG**  20:80 DMF:pH 10 buffer (v/v)  **YY**  pH 10 buffer | **Biotin-YG**  100 μL  **YY**  100 μL |  |  |  |

References

[1] a) L. Qian, X. R. Yang, *Adv. Funct. Mater.* **2007**, *17*, 1353; b) B. N. Bideh, A. Sousaraei, M. Moghadam, *Sci Rep.* **2024**, *14*, 16070; c) E. L. Sciuto, M. F. Santangelo, G. Villaggio, F. Sinatra, C. Bongiorno, G. Nicotra, S. Libertino, *Sens. Bio.-Sens. Res.* **2015**, *6*, 67; d) W. R. Kitzmann, K. Heinze, *Angew. Chem. Int. Ed.* **2022**, *62*, e202213207; e) E. G. Hohenstein, C. D. Sherrill, *J. Phys. Chem. A*, **2009**, *113*, 878.

[2] A. Barth. *Prog. Biophys. Mol. Biol*. **2000**, *74*, 141.

[3] H.-S. Jang, J.-H. Lee, Y.-S. Park, Y.-O. Kim, J. Park, T.-Y. Yang, K. Jin, J. Lee, S. Park, J. M. You, K.-W. Jeong, A. Shin, I.-S. Oh, M.-K. Kwon, Y.-I. Kim, H.-H. Cho, H. N. Han, Y. Kim, Y. H. Chang, S. R. Paik, K. T. Nam, Y.-S. Lee, *Nat. Commun*. **2014**, *5*, 3665.

[4] F. DeLange, C. H. Klaassen, S. E. Wallace-Williams, P. H. Bovee-Geurts, X. M. Liu, W. J. DeGrip, K. J. Rothschild, *J. Biol. Chem.* **1998**, *273*, 23735.

[5] C. Liu, J. Hua, P. F. Ng, Y. Wang, B. Fei, Z. Shao, *ACS Biomater. Sci. Eng.* **2022**, *8*, 484.

[6] J. L. Whittaker, N. R. Choudhury, N. K. Dutta, A. Zannettino, *J. Mater. Chem. B* **2014**, *2*, 6259.
